# Virulence and Stress Responses of *Shigella flexneri* Regulated by PhoP/PhoQ

**DOI:** 10.3389/fmicb.2017.02689

**Published:** 2018-01-15

**Authors:** Zhiwei Lin, Xia Cai, Mingliang Chen, Lina Ye, Yang Wu, Xiaofei Wang, Zhihui Lv, Yongpeng Shang, Di Qu

**Affiliations:** ^1^Key Laboratory of Medical Molecular Virology of Ministries of Education and Health, School of Basic Medical Science and Institutes of Biomedical Sciences, Shanghai Medical College of Fudan University, Shanghai, China; ^2^Shanghai Municipal Center for Disease Control and Prevention, Shanghai, China

**Keywords:** *S. flexneri*, two-component signal transduction system, PhoP/PhoQ, Mg^2+^, virulence, *icsA*

## Abstract

The two-component signal transduction system PhoP/PhoQ is an important regulator for stress responses and virulence in most Gram-negative bacteria, but characterization of PhoP/PhoQ in *Shigella* has not been thoroughly investigated. In the present study, we found that deletion of *phoPQ* (Δ*phoPQ*) from *Shigella flexneri* 2a 301 (*Sf*301) resulted in a significant decline (reduced by more than 15-fold) in invasion of HeLa cells and Caco-2 cells, and less inflammation (− or +) compared to *Sf*301 (+++) in the guinea pig Sereny test. In low Mg^2+^ (10 μM) medium or pH 5 medium, the Δ*phoPQ* strain exhibited a growth deficiency compared to *Sf*301. The Δ*phoPQ* strain was more sensitive than *Sf*301 to polymyxin B, an important antimicrobial agent for treating multi-resistant Gram-negative infections. By comparing the transcriptional profiles of Δ*phoPQ* and *Sf*301 using DNA microarrays, 117 differentially expressed genes (DEGs) were identified, which were involved in Mg^2+^ transport, lipopolysaccharide modification, acid resistance, bacterial virulence, respiratory, and energy metabolism. Based on the reported PhoP box motif [(T/G) GTTTA-5nt-(T/G) GTTTA], we screened 38 suspected PhoP target operons in *S. flexneri*, and 11 of them (*phoPQ, mgtA, slyB, yoaE, yrbL, icsA, yhiWX, rstA, hdeAB, pagP*, and *shf*–*rfbU*-*virK*-*msbB2*) were demonstrated to be PhoP-regulated genes based on electrophoretic mobility shift assays and β-galactosidase assays. One of these PhoP-regulated genes, *icsA*, is a well-known virulence factor in *S. flexneri*. In conclusion, our data suggest that the PhoP/PhoQ system modulates *S. flexneri* virulence (in an *icsA*-dependent manner) and stress responses of Mg^2+^, pH and antibacterial peptides.

## Introduction

*Shigella* is a facultative intracellular Gram-negative pathogen which causes shigellosis by penetrating and replicating within human colonic epithelial cells. This invasive process causes ulcerative lesions that result in a bloody and purulent diarrhea characteristic of bacillary dysentery. The bacteria are transmitted via the fecal-oral route and invade the mucosa of the colon. Based on the biotype, *Shigella* is divided into four subgroups and *Shigella flexneri* 2a is the main subgroup in China. *Shigella* is highly infectious and it causes shigellosis by infection with only 10 to 100 organisms (Watanabe, 1988). Due to the non-standard use of antibiotics and the spread of drug resistant genes, *Shigella* drug resistance in clinical settings is becoming more and more prominent, which makes it difficult for shigellosis clinical treatment (Benny et al., 2014; Chen et al., 2014; Zhang et al., 2014).

Bacterial infection of the hosts relies on the ability of bacteria to cope with the challenge of environmental pressures. The two-component system (TCS) is a multivariate regulation mechanism which widely exists in the prokaryotes and contributes to the adaptation of bacteria to environmental challenges. In these TCSs, the activation of a sensor histidine kinase leads to autophosphorylation and then transfers the phosphoryl group to the cognate response regulator, which further regulates the expression of downstream genes (Gooderham and Hancock, 2009; Singh et al., 2014). TCSs play important roles in *Shigella* virulence. For example, the OmpR/EnvZ TCS was reported to control the virulence of *S. flexneri* (Bernardini et al., 1990). It was noticed that the PhoP/PhoQ TCS is involved in virulence regulation in *S. typhimurium* (Miller et al., 1989; Perez et al., 2009). In 2000, Moss and coworkers showed that a *phoP* mutant decreased the inflammatory response and was more sensitive to polymorphonuclear leucocytes (PMNs) in *S. flexneri* (Moss et al., 2000), and our previous studies have shown that inhibitors of PhoQ reduced the virulence of *S. flexneri* (Cai et al., 2011). These results indicate that PhoP/PhoQ has the function of virulence regulation in *Shigella*, but the regulatory mechanism of PhoPQ in *Shigella* has not been confirmed.

PhoP/PhoQ is a broadly conserved TCS among many pathogenic and non-pathogenic bacteria. In most of these organisms the PhoPQ system has an original function of monitoring the extracellular Mg^2+^, while in pathogenic bacteria it also plays an important role in regulation of bacterial virulence phenotypes (Miller et al., 1989; Johnson et al., 2001; Grabenstein et al., 2006; Perez et al., 2009). The PhoP/PhoQ TCS consists of the histidine kinase PhoQ and the response regulator PhoP. PhoQ can respond to environmental signals by autophosphorylation. Phosphorylated PhoQ transfers the phosphate to PhoP, and activated PhoP further regulates the expression of downstream genes. Although the PhoP/PhoQ system has similar functions in regulating bacterial virulence in pathogenic bacteria such as *Salmonella typhimurium, Yersinia pestis* and *Mycobacterium tuberculosis* (Oyston et al., 2000; Cano et al., 2001; Perez et al., 2001), the regulons of PhoPQ vary in different species of bacteria. Groisman compared the PhoP-regulated genes in *Salmonella* and *E. Coli* and found that only a limited number of genes were in common between the two PhoP regulons (Groisman, 2001).

The virulence gene *icsA* (also named *virG*) is located on the virulence plasmid of *Shigella* and encodes the outer membrane protein IcsA, which is a key virulence factor in the *Shigella* pathogenesis (Bernardini et al., 1989). In the early stage of *Shigella* infection, the bacteria reach the intestinal lumen in which IcsA binds to the still unknown receptor to help *Shigella* adhere on the surface of the host cell (Brotcke Zumsteg et al., 2014). After invasion into the host cell, IcsA functions in activating the neural Wiskott-Aldrich syndrome protein (N-WASP) to mediate the intracellular actin-based motility (ABM), which is important for *Shigella* to survive within the host cell (Goldberg and Theriot, 1995). The *Shigella icsA* mutant strain shows a defect of bacterial intracellular actin assembly and cell-to-cell spread, followed by a significant decrease of virulence both in cells and animal models (Teh and Morona, 2013; Brotcke Zumsteg et al., 2014; Leupold et al., 2017).

In the present study, we have investigated the regulation functions of PhoP/PhoQ in *S. flexneri*. PhoPQ knocking out caused a decrease of *S. flexneri* virulence in HeLa cells and Caco-2 cells invasion models and guinea pig Sereny test, which was similar to that reported by Moss et al. (2000). The activity of PhoPQ allowed *Shigella* to tolerate low environmental Mg^2+^, acidic pH, and the presence of polymyxin B. Those environmental input signals promoted the expression of PhoPQ. We screened out 11 PhoP-regulated genes or operons in *Shigella* by using electrophoretic mobility shift assays (EMSAs) and β-galactosidase assays, in which a well-known virulence factor, *icsA*, was found and validated to be regulated by PhoPQ for the first time. It indicates that the PhoPQ system modulates *S. flexneri* virulence in an *icsA*-dependent manner.

## Materials and methods

### Ethics statement

All guinea pig infection procedures were approved by the Institutional Animal Care and Use Committee (IACUC) of School of Basic Medical Sciences, Fudan University (IACUC Animal Project Number 20140226-022-qu) according to national guidelines (Regulations for the Administration of Affairs Concerning Experimental Animals, China).

### Bacterial strains, plasmids, and growth conditions

*S. flexneri* 2a 301 (*Sf*301, GenBank accession number AE005674) was kindly provided by Pr. Qi Jin (MOH Key Laboratory of Systems Biology of Pathogens, Institute of Pathogen Biology, Chinese Academy of Medical Sciences and Peking Union Medical College, Beijing, China). The bacterial strains and plasmids used in this study are listed in Table 1. *S. flexneri* and *E. coli* were grown in Luria–Bertani medium (LB; Oxoid, Basingstoke, UK) at 37°C. Antibiotics were used at the following concentrations: ampicillin (100 μg/ml), kanamycin (50 μg/ml), tetracycline (10 μg/ml) and gentamicin (50 μg/ml) (Sigma-Aldrich, St. Louis, MO, USA).

**Table 1:** Bacterial strains and plasmids used in this study.

**Name**	**Description^a^**	**Source or references**
**BACTERIAL STRAINS**		
*Shigella*		
*Sf*301	Wild-type *S. flexneri* 2a 301	Jin et al., 2002
*ΔphoPQ*	*phoPQ* deletion of wild-type *Sf*301*, Kan*	This study
*ΔphoPQ*c	*ΔphoPQ* complemented with p*phoPQ, Amp, Kan*	This study
*ΔphoPQ*(pGEMT)	*ΔphoPQ* introduced with plasmid pGEMT	This study
*ΔphoPQ*(p*icsA*)	*ΔphoPQ* introduced with plasmid *picsA*	This study
*ΔicsA*	*icsA* deletion of wild-type *Sf*301*, Kan*	This study
*ΔicsAc*	*ΔicsA* complemented with p*icsA, Amp, Kan*	This study
*ΔicsA*(pGEMT)	*ΔicsA* introduced with plasmid pGEMT	This study
*Sf*301(p*phoP::lacZ*)	*Sf*301 introduced with plasmid p*phoP::lacZ*	This study
*Sf*301(p*shf::lacZ*)	*Sf*301 introduced with plasmid p*shf::lacZ*	This study
*Sf*301(p*icsA::lacZ*)	*Sf*301 introduced with plasmid p*icsA::lacZ*	This study
*Sf*301(p*lacZ*)	*Sf*301 introduced with plasmid p*lacZ*	This study
*ΔphoPQ*(p*phoP::lacZ*)	*ΔphoPQ* introduced with plasmid p*phoP::lacZ*	This study
*ΔphoPQ*(p*shf::lacZ*)	*ΔphoPQ* introduced with plasmid p*shf::lacZ*	This study
*ΔphoPQ*(p*icsA::lacZ*)	*ΔphoPQ* introduced with plasmid p*icsA::lacZ*	This study
*ΔphoPQ*(p*lacZ*)	*ΔphoPQ* introduced with plasmid p*lacZ*	This study
*E. coli*		
DH5α	*supE44 ΔlacU169 hsdR17 recA1 endA1 gyrA96 thi-1 relA1*	Invitrogen
BL21(DE3)	F-*ompT hsdS_*B*_*(r_B_-m_B_-) *gal dcm* (DE3)	Invitrogen
**PLASMIDS**		
pKD46	Red recombinase expression plasmids, low copy number, *Amp*	Datsenko and Wanner, 2000
pKD13	oriR6K, *Amp, Kan*	Datsenko and Wanner, 2000
pGEMT	PCR cloning vector, high copy number, *Amp*	Promega
p*phoPQ*	Wild-type gene *phoPQ* cloned into pGEMT	This study
p*icsA*	Wild-type gene *icsA* cloned into pGEMT	This study
pET28a	oriR, IPTG induced, *Kan*	Novagen
pET28a-*phoP*	pET28a with insertion of the gene *phoP*, for PhoP expression	This study
pBAD/His/LacZ	pBAD, LacZ ORF, pBR322 ori, *Amp*	Invitrogen
pACYC184	Medium copy number vector, p15A ori, *Cm, Tc*	Chang and Cohen, 1978
p*lacZ*	pACYC184 inserted with the promoterless lacZ gene PCR amplified from pBAD/His/LacZ	This study
p*phoP::lacZ*	p*lacZ* inserted with promoter region of *phoP*	This study
p*shf::lacZ*	p*lacZ* inserted with promoter region of *shf*	This study
p*icsA::lacZ*	p*lacZ* inserted with promoter region of *icsA*	This study

a *Kan, kanamycin resistance; Amp, ampicillin resistance; Cm, chloramphenicol resistance; Tc, tetracycline resistance*.

### Construction of *S. flexneri* deletion mutant and complementation strains

*S. flexneri phoQ/phoP* deletion mutant strain was constructed by one-step inactivation of chromosomal genes using PCR products (Datsenko and Wanner, 2000). First, *Sf*301 was transformed with pKD46 (Table 1) to express the λ Red recombinase and was selected by ampicillin. The transformants were induced with L-arabinose (1 mM) and made electro-competent cells. A *phoPQ* homologous recombination fragment with kanamycin resistance gene cassette (*kan*, 1,394 bp) was amplified from pKD13 with primers PhoPQus-kan-F/PhoPQds-kan-R (containing upstream and downstream regions of *Sf*301 *phoPQ*; Table 2). The purified PCR products were then digested with *Dpn*I, suspended in 10 mM Tris (pH 8.0), transferred into *Sf*301 by electroporation, and grown at 30°C. The *phoPQ* knockout mutant was screened out by kanamycin, and verified by PCR and sequencing (Sangon Biotech, Shanghai, China). The *phoPQ* knockout mutant was grown at 43°C to remove pKD46, plasmid removal was confirmed by PCR, and the strain was named Δ*phoPQ* (Figure S1).

**Table 2:** Primers used in this study.

**Primer^a^**	**Sequence (5′−3′)**	**Location (bp)^b^**	**Product length (bp)**	**Annotation^c^**
**CONSTRUCTION AND IDENTIFICATION OF *ΔphoPQ***				
PhoPQus-kan-F	AATCGCGTTACACTATTTTAATAATTAAGACAGGGAGAAATAAAAGTGTAGGCTGGAGCTGCTTCG	1191632–1191676		Underline: up and downstream
PhoPQds-kan-R	GAATCAATGACTTGATGTAGTGGTAAAAGGACATATTTATTCATCATTCCGGGGATCCGTCGACC	1189464–1189508	1,394	regions of *phoPQ*
InterPQ-F	CTGGTTGTTGAAGACAATGCG	1191602–1191622	2,087	
InterPQ-R	AATCACCTCCATCCGCGCACC	1189536–1189556		
OuterPQ-F	AATCAGTGCCGGATGGCGATG	1191773–1191793	2,413	
OuterPQ-R	TTCATACAGTGCACCGAACGG	1189381–1189401		
pKD46-F	GCAGAACACATCCGGTACATG	1592–1612	641	
pKD46-R	CTGACGTTCTGCAGTGTATGC	2212–2232		
**CONSTRUCTION OF *ΔphoPQ*c**				
*ΔphoPQ*c-F	GCCTCAAATCAGTGCCGGATG	1191779–1191799	2,414	
*ΔphoPQ*c-R	ACAGTGCACCGAACGGTGTAG	1189386–1189406		
**CONSTRUCTION OF pET28a-*****phoP***				
pET28a-phoP-F	CGCGGATCCATGCGCGTACTGGTTGTTGAA	1191611–1191631	669	Underline: BamHI
pET28a-phoP-R	CCGCTCGAGGCGCAATTCGAACAGGTAGCC	1190963–1190983		Underline: XhoI
**AMPLIFICATION OF GENE PROMOTER REGIONS**				
P_phoP_-F	GCCTCAAATCAGTGCCGGATG	1191779–1191799	176	
P_phoP_-R	ACGCGCATTTTTATTTCTCCC	1191624–1191644		
P_mgtA_-F	CTGTTGTCCCATAACGTGTTG	4419849–4419869	187	
P_mgtA_-R	CCATATAACCTCCGGTAAGTG	4419683–4419703		
P_slyB_-F	CGTGAATACCATGCGGAATGA	1697757–1697777	186	
P_slyB_-R	AGCATCCCTCATGGTCAAAGT	1697922–1697942		
P_yoaE_-F	GATCCGTAATTTAACTTTCGA	1448408–1448428	214	
P_yoaE_-R	AGAAAGGCAGGCGTTAAAAGG	1448601–1448621		
P_rstA_-F	GTGGAATCAGCCCGGCGATAT	1656568–1656588	220	
P_rstA_-R	CGGTAGATATAAAAACGTCAC	1656767–1656787		
P_shf_-F	GAGTACCTGTGTTGTTCTGAG	191446–191466	224	
P_shf_-R	AACCCAATAAAGCTGGTGCAT	191649–191669		
P_icsA_-F	TTATCGAACATATAGCTTTCC	149445–149465	189	
P_icsA_-R	ATCAGTAAGTGGTTGATAAAC	149613–149633		
P_hdeA_-F	ATCCCCTGCTATCAATCTATG	3634002–3634022	209	
P_hdeA_-R	TAAAGTGAAAGAGCCGTCACG	3633814–3633834		
P_yrbL_-F	AATCACGTACTGAAATCGTTC	3340458–3340478	168	
P_yrbL_-R	GAATCATGCCATCTCCTGGAA	3340605–3340625		
P_yhiW_-F	GGAAACTTTGTGCTCTCAGTA	3698224–3698244	244	
P_yhiW_-R	CTGCGATTATTTCAATTTCAG	3698447–3698467		
P_pagP_-F	AGATGATTGTTGTATCTCGTA	694218–694238	246	
P_pagP_-R	TCTACTACTAGCATAGCAAAG	693993–694013		
P_ipaH7.8_-F	CCTCTGGAGCTTTATCCAGTC	61790–61810	210	
P_ipaH7.8_-R	AGGAAATGTAAGCCGAGTAAG	61979–61999		
P_virA_-F	TTCTGTACGCTTGCCCAAAGT	149370–149390	203	
P_virA_-R	ATGGAATGTTATTCTTCTCTT	149188–149208		
**CONSTRUCTION OF THE LacZ FUSION**				
lacZ-F	AAAAGTACTGACGATGACGATAAGGATCCA		3,102	Underline: ScaI
lacZ-R	CATGCCATGGCATCCGCCAAAACAGCCAAGC			Underline: NcoI
P_phoP_-lacZ-F	TCCCTCGGGGCCTCAAATCAGTGCCGGATG	1191779–1191799	176	Underline: AvaI
P_phoP_-lacZ-R	AAAAGTACTACGCGCATTTTTATTTCTCCC	1191624–1191644		Underline: ScaI
P_shf_-lacZ-F	TCCCTCGGGTTATCGAACATATAGCTTTCC	191446–191466	224	Underline: AvaI
P_shf_-lacZ-R	AAAAGTACTATCAGTAAGTGGTTGATAAAC	191649–191669		Underline: ScaI
P_icsA_-lacZ-F	TCCCTCGGGGAGTACCTGTGTTGTTCTGAG	149445–149465	189	Underline: AvaI
P_icsA_-lacZ-R	AAAAGTACTAACCCAATAAAGCTGGTGCAT	149613–149633		Underline: ScaI

a *Primers were designed according to the genomic sequence of S. flexneri 2a 301 (GenBank accession number AE005674). F, forward primer; R, reverse primer*.

b *Location is the locus of the primer in the genomic sequence of S. flexneri 2a 301*.

c *Underlined sequences represent the upstream and downstream regions of phoPQ or restriction enzyme sites*.

For construction of complementation of *phoPQ* for Δ*phoPQ*, the *phoPQ* operon with its promoter region (2,414 bp) was amplified by PCR with primers Δ*phoPQ*c-F/Δ*phoPQ*c-R (Table 2), which were designed based on the genome of *Sf*301. The PCR products were ligated with pGEMT (Table 1), and the bacteria with the insertion were selected by ampicillin. After verification by sequencing, the *phoPQ* complementary plasmid (p*phoPQ*) was transformed into Δ*phoPQ*, and the transformants were selected by kanamycin and ampicillin, verified by PCR and sequencing, and named Δ*phoPQ*c. pGEMT was introduced into Δ*phoPQ* as a vector control, and was named Δ*phoPQ* (pGEMT).

The construction of *S. flexneri icsA, yoaE, yrbL*, or *rstA* deletion mutant strains and their complementation strains used the same method as that of *phoPQ* with primers listed in Table S4. For construction of the *icsA* complementary plasmid p*icsA*, the *icsA* gene with its promoter region (3,642 bp) was amplified from wild-type *Sf*301 with primers Δ*icsA*c-F/Δ*icsA*c-R, and ligated into the pGEMT vector. After verification by sequencing, p*icsA* was then transformed into Δ*phoPQ* to construct the *icsA* expression strain Δ*phoPQ*(p*icsA*), verified by PCR and sequencing.

### Invasion assay with *S. flexneri*

The invasion ability of strains of *S. flexneri* was determined by gentamicin protection assay on HeLa cells and Caco-2 cells (Hale and Formal, 1981; Mounier et al., 1992). Cells were grown in 24-well plates at 5% CO_2_ [HeLa cells in Dulbecco's Modified Eagle Medium (DMEM) with 10% fetal bovine serum (FBS) and Caco-2 cells in DMEM medium with 20% FBS]. Bacterial strains were inoculated into LB containing 0.3 M NaCl until the OD_600_ reached 1.0. Bacteria were added to semi-confluent HeLa cells or Caco-2 cells at a multiplicity of infection (MOI) of 10. The plates were then centrifuged at 900 g for 10 min. After incubating at 37°C for 5, 15, or 30 min, gentamicin was added to the medium with a final concentration of 100 μg/ml for 60 min at 37°C. The cells were then lysed with 1 ml 0.1% Triton X-100 in PBS for 10 min. The lysates were diluted 1:10 in PBS and plated onto LB agar plates in triplicate, and colonies were counted. The invasion rate was calculated as the number of bacteria recovered from gentamicin-treated cells divided by the total number of inoculated bacteria.

For immunofluorescence assay, HeLa cells grown in 24-well tissue culture plates with coverslips were infected with strains of *S. flexneri* for 15 min, fixed with 3.7% formaldehyde in PBS for 15 min, and then permeabilized with 0.2% Triton X-100 for 5 min. Bacteria were stained with anti-*Shigella* O-Ag serum (BD Biosciences, New York, USA) and then with IgG Alexa 488 conjugate (Life Technologies, New York, USA), actin was stained with Texas Red phalloidin (Life Technologies), and nuclei were stained with 4′,6-Diamidino-2-Phenylindole (DAPI, Life Technologies) for 30 min. The coverslips were mounted and observed under a confocal laser scanning microscope (CLSM; Leica TCSSP5, Mannheim, Germany) at × 100 magnification (Chang et al., 2005).

### *S. flexneri* sereny test and pathological examination

The virulence of *S. flexneri* was determined by guinea pig keratoconjunctivitis Sereny test (Sereny, 1957). Female guinea pigs (age 6 weeks, about 300 g) were inoculated with 10^9^ Colony-Forming Units (CFU) of bacteria per eye (six guinea pig eyes in each group), and observed at 24, 48, and 72 h. Inoculation with LB served as a negative control. The keratoconjunctivitis induced by the bacteria was scored as follows: −, no disease or mild irritation; +, mild conjunctivitis or late development and/or rapid clearing of symptoms; ++, keratoconjunctivitis without purulence; and +++, fully developed keratoconjunctivitis with purulence. At 72 h post-inoculation, guinea pigs were euthanized with pentobarbital (40 mg/kg) and the eyes were removed and fixed in 4% formalin in PBS (pH 7.2). After hematoxylin and eosin (H&E) staining, the eye sections were examined under a microscope.

### Bacterial growth curves under low Mg^2+^, acidic pH and the presence of polymyxin B conditions

Growth curves of the strains were determined by measuring the OD_600_ with an Eppendorf spectrophotometer at 60 min intervals over a period of 14 h. For the low Mg^2+^ growth assay, N medium was used containing 0.1 M Tris-HCl (pH 7.4), 38 mM glycerol, 0.1% (wt/vol) Casamino Acids, 0.37 g/l KCl, 0.087 g/l K_2_SO_4_, 0.99 g/l (NH_4_)_2_SO_4_ and 0.14 g/l KH_2_PO_4_ (Barchiesi et al., 2012). Overnight cultures of bacterial strains were inoculated into N medium supplemented with 10 μM or 10 mM MgCl_2_ (at 1:50 dilution) and incubated at 37°C with shaking. To assay acid resistance of bacteria, E glucose broth was used containing 0.2 g/l MgSO_4_•7H_2_O, 2 g/l citric acid, 13.1 g/l K_2_HPO_4_•3H_2_O, 3.5 g/l Na(NH_4_) HPO_4_•4H_2_O, and 0.4% glucose. Overnight cultures were inoculated into E glucose broth at pH 7 or pH 5 (at 1:50 dilution) and incubated at 37°C with shaking (Barchiesi et al., 2012). For the polymyxin B resistance assay, overnight cultures were inoculated into LB, grown with shaking until the OD_600_ reached 0.6, then bacteria were diluted in sterile 0.85% saline to about 5 × 10^3^ cells per ml and exposed into different concentrations of polymyxin B (5, 10, 20, and 40 μg/ml) for 1 h at 37°C. Surviving bacteria were determined by plating on LB agar plates in triplicate. The survival rate was calculated as the number of bacteria treated with polymyxin B divided by that of the untreated control. All experiments were repeated at least three times.

### Microarray analysis and qRT-PCR

For microarray analysis, *Sf*301 and Δ*phoPQ* were inoculated into LB medium and grown to mid-log phase (6 h) or early-stationary phase (10 h), with three biological replicates. Cells were harvested by centrifugation at 10,000 g for 1 min, and total RNAs were extracted using the RNeasyH Mini Kit (QIAGEN, Hilden, Germany) following the manufacturer's instructions. Agilent custom-specific design GeneChip of *Sf*301 genomic DNA were used. Each microarray (4^*^44k) contained spots with 4168 specific 60-mer oligonucleotides representing the 4168 ORFs of *Sf*301 in triplicate, carried out by Shanghai Biotechnology Co. Ltd. (Shanghai, China) according to standard protocols provided by Agilent Technologies (Palo Alto, USA). Briefly, the quality and quantity of RNA samples were determined and checked by Agilent 2100 bioanalyzer (Agilent Technologies). The RNA samples were then reverse transcribed to cDNA by MMLV reverse transcriptase (Invitrogen, Carlsbad, USA), followed by transcription with T7 RNA polymerase (New England BioLabs, Beverly, UK) to generate aminoacyl-UTP-labeled cRNA. Amino allyl modified cRNAs were purified and labeled with Cy3 (Cy3 NHS ester, GE Healthcare, Piscataway, NJ). Labeled cRNAs were then fragmented in fragmentation buffer (Agilent Technologies) and mixed with the Gene Expression Hybridization Kit (Agilent Technologies) at 65°C for 17 h with a constant rotation rate of 10 rpm for hybridization. The arrays were scanned by an Agilent DNA Microarray scanner. Microarray data were normalized in the Agilent Feature Extraction software. The ratio of gene expression (Δ*phoPQ* vs. *Sf*301) was calculated from the normalized signal intensities. A false discovery rate of 5% (*P*-value cutoff; 0.05) was used for variance analysis of three biological replicates and an arbitrary threshold of 2.0-fold or 0.5-fold was used for defining significant differences in expression ratios.

For validating the differential expression genes of microarray, qRT-PCR was carried out. Total RNA of bacteria was extracted using the RNeasyH Mini Kit. The extracted RNA was reverse transcribed into cDNA using iScript reverse transcriptase (Bio-Rad, Hercules, CA, USA) with incubation for 5 min at 25°C, followed by 30 min at 42°C and 5 min at 85°C. Subsequently, qRT-PCRs were performed using SYBR green PCR reagents (Premix EX TaqTM, Takara Biotechnology, Dalian, China) in the Mastercycler realplex system (Eppendorf AG, Hamburg, Germany) with amplification conditions of 95°C for 30 s, 40 cycles of 95°C for 5 s and 60°C for 34 s, followed by melting curve analysis. The 16S rRNA methyltransferase coding gene *rsmC* was used to normalize the transcriptional levels of genes in the qRT-PCRs. All qRT-PCRs were carried out in triplicate with at least three independent RNA samples. The primers (Table S1) were designed based on the genome of *Sf*301 using Beacon designer software (Premier Biosoft International Ltd., Palo Alto, CA, USA).

### EMSA

For analyzing the interaction of the recombinant PhoP and the promoter regions of putative target genes, EMSA were performed using the DIG Gel Shift Kit (Roche, Basel, Switzerland) according to the manufacturer's instructions. The *phoP* gene was amplified with primers pET28a-phoP-F/pET28a-phoP-R (Table 2) from the genomic DNA of *Sf*301 and inserted into the vector pET-28a (+) to obtain the recombinant plasmid pET28a-*phoP*. The recombinant plasmid was then transformed into *Escherichia coli* BL21 (DE3). Bacteria were grown to an OD_600_ of 0.6 at 37°C and 0.8 mM IPTG was then added to induce PhoP protein expression for 6 h at 30°C. The expressed His-tagged PhoP protein was purified using the ProBondTM Purification System (Invitrogen, Carlsbad, CA, USA) according to the manufacturer's instructions. PhoP was phosphorylated prior to gel shift reaction by incubating PhoP with 50 mM acetylphosphate for 1 h. The predicted promoter regions of putative target genes were amplified with primers in Table 2 and labeled with digoxigenin using terminal transferase. Each gel shift assay included the probe labeled with digoxigenin plus increasing concentrations of the phosphorylated PhoP (PhoP-P, ranging from 0.16 to 1.6 μM). The coding sequence of *virA* in *Sf*301 without PhoP box sequence was designated as a negative control. All samples were incubated at 25°C for 20 min, separated by electrophoresis on 6% non-denaturing polyacrylamide gel, blotted onto a positively charged nylon membrane (Millipore), and detected by an enzyme immunoassay following the manufacturer's instructions.

### DNase I footprinting assay

DNase I footprinting assays were performed according to Wang et al. (2012). The promoter regions of *yoaE, mgtA* and *shf* were amplified with primers listed in Table 2, and separately cloned into the pUC18BT vector (Shanghai Biotechnology Corporation, China), which was further used as the template for preparation of fluorescent FAM labeled probes. The probes were purified by the Wizard® SV Gel and PCR Clean-Up System (Promega) and quantified with a NanoDrop 2000C (Thermo Fisher Scientific Waltham, MA, USA). For each assay, 500 ng probes were incubated with different amounts of PhoP in 40 μl binding buffer at 30°C for 30 min. Then 10 μl DNase I (0.01 unit) (Promega) and 100 nmol CaCl_2_ were added, incubated at 25°C for 1 min, and the reactions were stopped by adding 140 μl DNase I stopping solution (200 mM unbuffered sodium acetate, 30 mM EDTA and 0.15% SDS). The DNA samples were extracted with phenol/chloroform, dissolved in 30 μl MilliQ water and loaded for carrying out capillary electrophoresis. The data were collected using the GeneScan-500 LIZ dye Size Standard (Applied Biosystems, Foster City, CA, USA).

### LacZ fusion and β-galactosidase assay

A promoterless *lacZ* reporter gene was amplified by PCR with primers lacZ-F/lacZ-R (Table 2) using the pBAD/His/LacZ vector (Invitrogen, Carlsbad, CA, USA) as a template. Purified PCR products were digested with ScaI and NcoI endonucleases (MBI Fermentas, Vilnius, Lithuania) and inserted into the medium-copy-number plasmid pACYC184 (Chang and Cohen, 1978). The bacteria with the insertion were selected using tetracycline, verified by PCR and sequencing, and designated p*lacZ*. The promoter-proximal DNA region of *phoP, shf*, and *icsA* were amplified by PCR with Pfu DNA polymerase (Takara Biotechnology, Dalian, China) using the primers listed in Table 2. PCR products were digested with AvaI and ScaI endonucleases, and inserted into plasmid p*lacZ*. The bacteria with the insertion were selected by tetracycline, verified by PCR and sequencing, and named p*phoP::lacZ*, p*shf::lacZ*, and p*icsA::lacZ*. These recombinant plasmids were introduced into *Sf*301 and the Δ*phoPQ* strain, respectively. The promoterless p*lacZ* was transferred into the bacterial strains as negative control. The reporter bacterial strains were separately grown in LB, or N medium with 10 μM/10 mM MgCl_2_, or E glucose broth at pH 7/5.5 or LB with 25 μg/ml polymyxin B. The bacteria were harvested, lysed with 400 μl lysozyme (1 mg/ml) at 37°C for 30 min, and then the β-galactosidase activity in the cellular extracts was measured by the β-Galactosidase Enzyme Assay System (Promega) following the manufacturer's instructions. All experiments were repeated at least three times independently.

### Statistical analysis

Experiments were performed in triplicate and repeated at least three times. The data were analyzed with Student's *t*-test or one-way factorial analysis of variance in SPSS version 14.0 (Chicago, IL). Differences in means with a *P* < 0.05 were considered significant.

### Microarray accession number

The complete microarray data set is uploaded in the Gene Expression Omnibus database (http://www.ncbi.nlm.nih.gov/geo/) under accession numbers GPL24308 for the platform design and GSE107365 for the original data set.

## Results

### Deletion of *phoPQ* diminished *S. flexneri* virulence

To analyze the regulatory mechanism of PhoPQ in *Shigella*, a *S. flexneri phoPQ* deletion mutant strain was constructed by using homologous recombination (Datsenko and Wanner, 2000). After transformation of the λ Red recombinase expression plasmid pKD46, the *Sf*301 was transformed with a fragment of a kanamycin resistance cassette with long flanking regions homologous of *phoPQ*. The *phoPQ* knockout mutant was then screened out by kanamycin, verified by PCR and sequencing, pKD46 was removed by growth at 43°C, and the strain named as Δ*phoPQ* (Figure S1). For construction of the *phoPQ* complemented strain, the *phoPQ* operon with its promoter region was amplified from wild-type *Sf*301, and ligated into the pGEMT vector. The *phoPQ* expression plasmid was then transformed into Δ*phoPQ*, selected by growth in the presence of kanamycin and ampicillin, verified by PCR as well as sequencing, and named as Δ*phoPQc*.

**Figure 1:**
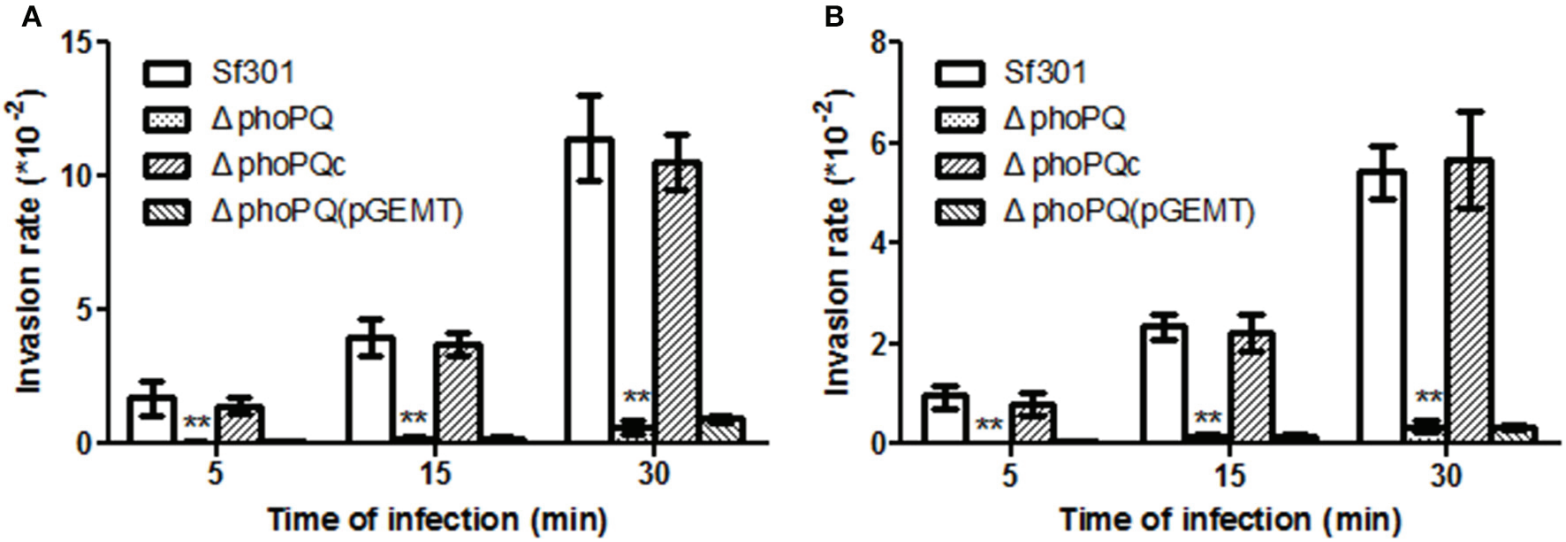
The invasion ability of *Sf*301, Δ*phoPQ* and Δ*phoPQc* in HeLa cells and Caco-2 cells. The gentamicin protection assay was used as a cellular model to evaluate the effect of *phoPQ* deletion on virulence of *Shigella*. Bacteria grown to logarithmic phase were added into the cells for 5, 15, or 30 min. Then gentamicin was added into the medium to kill extracellular bacteria. Colonies of lysates on LB plates were counted. The invasion rate is calculated as the number of intracellular bacteria divided by the number of inoculated bacteria and multiplied by 10,000. The Δ*phoPQ*(pGEMT) was used as an empty plasmid control. **(A)** Bacterial ability to invade HeLa cells. **(B)** Bacterial ability to invade Caco-2 cells. Values are means ± standard deviations from three independent wells. ^**^P < 0.01.

**Figure 2:**
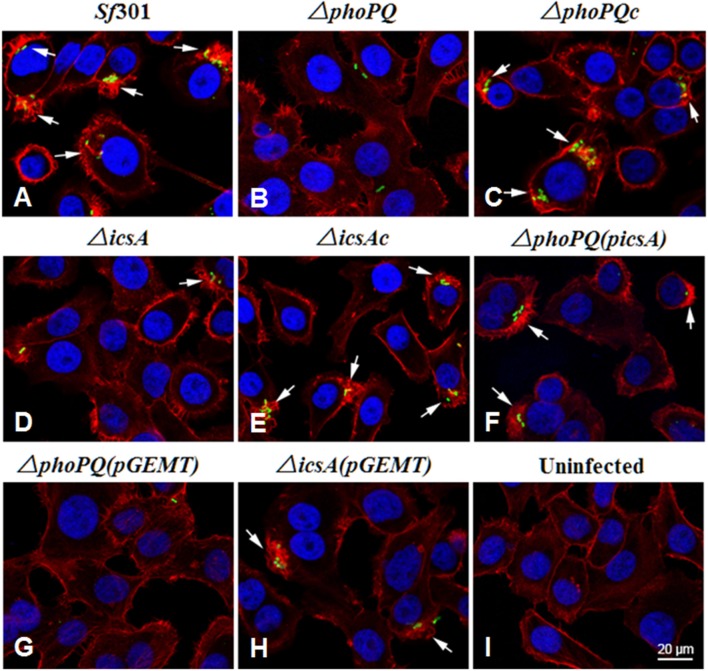
Changes in the cytoskeleton of HeLa cells infected with *S. flexneri* strains. HeLa cells were infected with *S. flexneri* strains for 15 min. Actin was visualized by staining with Texas Red-labeled phalloidin (red), bacteria were stained with rabbit polyclonal anti-*Shigella* anti-serum (green), and nuclei of HeLa cells and bacterial DNA were stained with DAPI (blue). The coverslips were mounted and observed under a confocal laser scanning microscope at ×100 magnification **(A–I)**. Arrows indicate locations of membrane ruffles. The Δ*phoPQ*(pGEMT) and Δ*icsA*(pGEMT) were used as empty plasmid controls.

The invasion ability of *Sf*301, Δ*phoPQ* and Δ*phoPQc* were evaluated by the gentamicin protection assay on HeLa cells and Caco-2 cells. Bacterial strains were separately inoculated into HeLa cells or Caco-2 cells at a MOI of 10, incubated for 5, 15, or 30 min, and then gentamicin was added to kill extracellular bacteria. In HeLa cells, invasion rates of Δ*phoPQ* at 5, 15, or 30 min post-inoculation were reduced by 56-fold, 22-fold, and 15-fold, respectively, compared with those of *Sf*301. The invasion ability of Δ*phoPQc* (complementary *phoPQ*) was recovered to levels of *Sf*301 (Figure 1A). In Caco-2 cells, invasion rates of Δ*phoPQ* were similar to those in HeLa cells (Figure 1B). Next, *Shigella*-infected HeLa cells were observed by confocal immunofluorescence microscopy. HeLa cells grown on coverslips in 24-well tissue culture plates were infected with the strains of *S. flexneri* at a MOI of 10 and incubated for 15 min. The infected cells were stained with rabbit polyclonal anti-*shigella* anti-serum, Texas Red-labeled phalloidin, and DAPI (Figure 2). In *Sf*301 infected cells, more cells displayed membrane ruffling (more than 5 cells showing obvious membrane ruffling in a microscope field of view at ×100 magnification), which indicated actin cytoskeleton changes of HeLa cells, while in Δ*phoPQ* infected cells no obvious membrane ruffling observed. The membrane ruffling of Δ*phoPQc* infected cells could be restored to the level of *Sf*301 infected cells by complementation with p*phoPQ* (Figures 2A–C).

**Figure 3:**
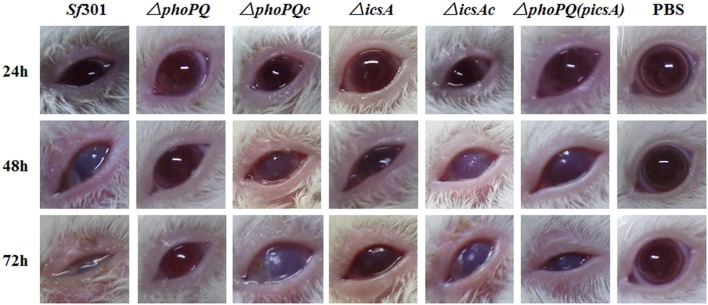
Keratoconjunctivitis in guinea pig eyes produced by *S. flexneri* strains. Female guinea pigs (age 6 weeks, about 300 g) were inoculated with 10^9^ CFU of bacteria per eye (six guinea pig eyes in each group), and observed at 24, 48, and 72 h. Guinea pig eye inoculation with PBS served as a negative control.

**Figure 4:**
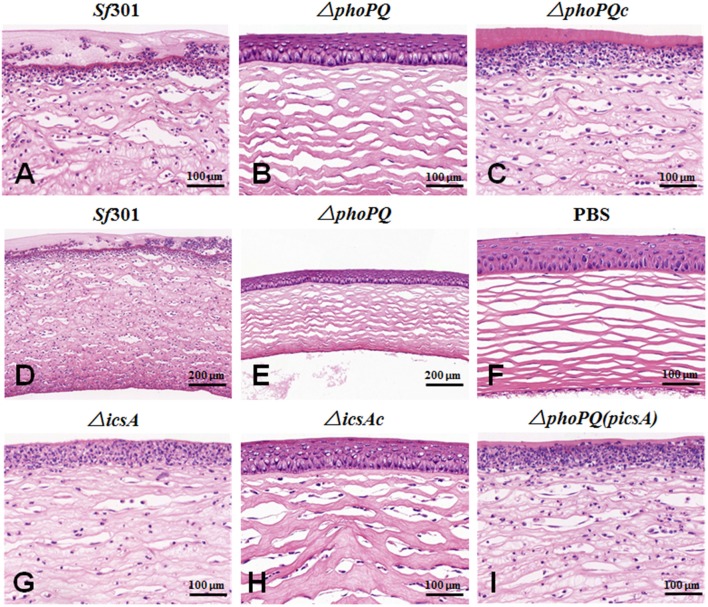
Pathological examination of guinea pig eyes infected with *S. flexneri* strains in Sereny test. Guinea pigs of the Sereny test were euthanized at 72 h post-infection and the eyes were removed and fixed in 4% formalin in PBS (pH 7.2). After hematoxylin and eosin (H&E) staining, eye sections were examined using a light microscope at ×400 **(A–I)** or ×200 **(D,E)** magnification.

The Guinea pig Sereny test was used to determine the virulence of *Sf*301, Δ*phoPQ*, and Δ*phoPQc*. Guinea pigs were infected with 10^9^ CFU per eye and observed at different time points. At 24 h post-infection, the guinea pig eyes infected with *Sf*301 displayed keratoconjunctivitis with or without purulence (+ or ++). At 48 and 72 h post-infection, the inflammation exacerbated with great purulence (+++). The Δ*phoPQ*-infected guinea pig eyes displayed no obvious inflammation (–) at 24 h post-infection, and a slight keratoconjunctivitis without purulence (+) after 48 h. In Δ*phoPQc*-infected guinea pig eyes, the inflammation reaction was similar to that of *Sf*301. Guinea pigs inoculated with PBS were used as a negative control (Figure 3, Table 3). At 72 h post-infection, all guinea pigs were euthanized with pentobarbital and the eyes were removed, stained with hematoxylin and eosin and pathological examinations were carried out. *Sf*301-infected eyes showed typical inflammatory reactions including luminal debris, epithelial desquamation, neutrophil infiltration and submucosal edema along with severe ulcers (Figures 4A,D). In contrast, Δ*phoPQ*-infected eyes showed very small numbers of neutrophils in the mucosal lamina or submucosa, and minimal edema in the submucosa (Figures 4B,E). The inflammatory lesions induced by Δ*phoPQc* recovered to the *Sf*301 level (Figure 4C).

**Table 3:** Degree of keratoconjunctival inflammation in guinea pig eyes infected with *S. flexneri* strains.

**Bacterial strain**	**24 h**						**48 h**						**72 h**					
	**1**	**2**	**3**	**4**	**5**	**6**	**1**	**2**	**3**	**4**	**5**	**6**	**1**	**2**	**3**	**4**	**5**	**6**
*Sf*301	++	+	++	+	++	+	+++	+++	+++	+++	+++	++	+++	+++	+++	+++	+++	+++
*ΔphoPQ*	–	–	–	–	–	–	–	–	+	–	–	–	–	+	+	–	+	–
*ΔphoPQ*c	++	+	+	+	+	++	+++	+++	++	++	+++	+++	+++	+++	++	+++	+++	+++
*ΔicsA*	–	+	+	–	+	–	+	+	+	–	+	+	+	++	+	–	+	++
*ΔicsAc*	++	++	+	++	+	+	+++	+++	++	+++	++	++	+++	+++	++	+++	+++	++
*ΔphoPQ*(p*icsA*)	+	++	+	+	+	+	++	++	++	++	++	+	++	+++	++	++	+++	++
PBS	–	–	–	–	–	–	–	–	–	–	–	–	–	–	–	–	–	–

*The degree of keratoconjunctival inflammation in each of the six guinea pigs infected with S. flexneri strains and PBS at 24, 48, and 72 h (n = 6). Guinea pig keratoconjunctivitis test was rated as follows: –, no disease or mild irritation; +, mild conjunctivitis or late development and/or rapid clearing of symptoms; ++, keratoconjunctivitis without purulence; and +++, fully developed keratoconjunctivitis with purulence*.

### Deletion of *phoPQ* decreased the ability of *S. flexneri* to withstand the challenge of environmental stress

**Figure 5:**
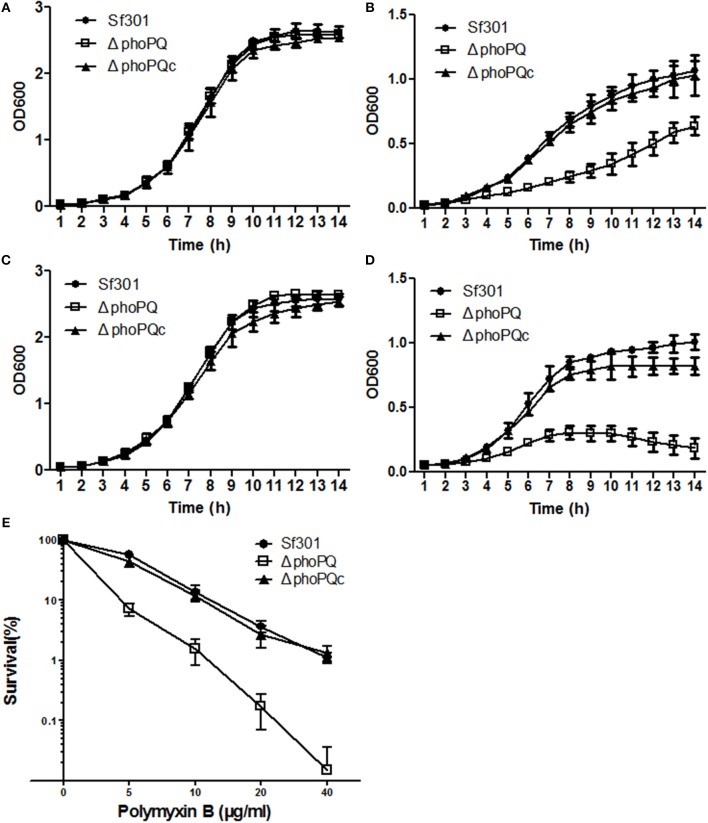
PhoPQ is required for *Shigella* to resist an environment of low Mg^2+^, acidic pH, or the presence of polymyxin B. *Sf*301, Δ*phoPQ* and Δ*phoPQc* were grown in N minimal medium supplemented with 10 mM MgCl_2_ **(A)** or 10 μM MgCl_2_ **(B)**. The OD_600_ was determined every 60 min for 14 h. *Sf*301, Δ*phoPQ* and Δ*phoPQc* were grown in E glucose broth at pH 7 **(C)** or pH 5 **(D)**. The OD_600_ was determined every 60 min for 14 h. *Sf*301, Δ*phoPQ* and Δ*phoPQc* were diluted in sterile 0.85% saline to about 5 × 10^3^ cells per ml, and exposed for 1 h at 37°C to various concentrations of polymyxin B. Survivors were measured by plating on LB. The survival rate was calculated as the number of bacteria treated with polymyxin B divided by that of the untreated control **(E)**. Results are mean from three independent assays performed in duplicate. Error bars correspond to standard deviations.

Extracellular Mg^2+^, pH and antimicrobial peptides have been described as input signals of the PhoP/PhoQ system (Garcia Vescovi et al., 1996; Gunn and Miller, 1996; Bearson et al., 1998; Groisman, 2001; Lejona et al., 2003; Barchiesi et al., 2012). Therefore, the role of PhoPQ in *S. flexneri* ability to withstand low Mg^2+^, acidic pH, or the presence of polymyxin B was analyzed. *Sf*301, Δ*phoPQ* and Δ*phoPQc* were grown in different conditions and growth curves were determined. In high Mg^2+^ medium (10 mM), the three strains showed no difference in growth pattern (Figure 5A), while in low Mg^2+^ medium (10 μM), Δ*phoPQ* showed limited growth compared to that of *Sf*301. At mid-log phase (8 h), *Sf*301 reached an OD_600_ of 0.68, but Δ*phoPQ* only reached an OD_600_ of 0.25 and took about 6 h more to reach an OD_600_ of 0.64. This growth deficiency was rescued by complementation with p*phoPQ* (Figure 5B). In neutral medium (pH 7), growth curves showed no difference among the three strains (Figure 5C). However, in the acidic medium (pH 5), *Sf*301 and Δ*phoPQ*c showed only limited growth while Δ*phoPQ* was unable to grow (Figure 5D). At different concentrations of polymyxin B (5, 10, 20, 40 μg/ml), the survival rates of Δ*phoPQ* were significantly lower than that of *Sf*301 (*P* < 0.05), and the resistance defect of Δ*phoPQ* was restored by complementation with p*phoPQ* (Figure 5E).

### Identification of PhoP-regulated genes in *S. flexneri*

DNA microarray was used to compare the transcriptional profiles of *Sf*301 and Δ*phoPQ* at middle-log phase (6 h) or early-stationary phase (10 h) under LB growth conditions. At middle-log phase, 117 differentially expressed genes (DEGs) were identified. Among them, 32 genes were up-regulated and 85 genes were down-regulated in Δ*phoPQ* (Table 4). At early-stationary phase, 54 DEGs were identified, with 19 genes up-regulated and 35 genes down-regulated in Δ*phoPQ* (Table S5). The 117 DEGs were involved in metal ion transport (*katE, narU, bfr*), acid resistance (*hdeABCD, gadAB, yhiWX, xasA*), lipopolysaccharides (LPS) modification and antibacterial peptide tolerance (*rfbU, mdoB, slyB, pagP, msbB2, pmrD*), signal transduction (*phoPQ, rstA, cstA*), bacterial virulence (*icsA, virK*), respiratory and energy metabolism (*hyaABCDEF, appABC*) (Table 4). Among them, 44 DEGs were verified by qRT-PCR, with 38 giving a result consistent with that of the DNA microarray (Table 4).

**Table 4:** Differentially expressed genes of Δ*phoPQ* compared to *Sf*301 by microarray and qRT-PCR at middle-log phase.

**Gene**	**Expression ratio (mutant/WT)^a^**			**Location**	**Description or predicted function**
	**Microarray^b^**	***P*-values^c^**	**qRT-PCR^d^**		
**AMINO ACID TRANSPORT AND METABOLISM**					
*gntT*	0.09	<0.0001	ND	Chromosome	High-affinity transport permease for gluconate
*tdcC*	3.19	0.0224	1.01 ± 0.21	Chromosome	Threonine/serine transporter TdcC
*edd*	0.27	0.0011	ND	Chromosome	Phosphogluconate dehydratase
*nanA*	0.27	0.0129	0.45 ± 0.13	Chromosome	*N*-Acetylneuraminate lyase
*exuT*	0.32	0.0252	ND	Chromosome	Transport protein of hexuronates
*ybaS*	0.32	0.0002	0.15 ± 0.04	Chromosome	Glutaminase
*gntU*	0.33	0.0002	ND	Chromosome	Low affinity gluconate transporter
*xasA*	0.35	0.0227	0.48 ± 0.01	Chromosome	Acid sensitivity protein, putative transporter
*ybaT*	0.39	0.0006	ND	Chromosome	Putative amino acid/amine transport protein
*ggt*	0.41	0.0001	ND	Chromosome	Gamma-glutamyltranspeptidase
*poxB*	0.41	0.0011	ND	Chromosome	Pyruvate dehydrogenase
*sdaA*	0.44	0.0499	ND	Chromosome	L-serine deaminase
*ybdR*	0.44	0.0005	0.68 ± 0.12	Chromosome	Putative oxidoreductase
*nanT*	0.48	0.0297	ND	Chromosome	Putative sialic acid transporter
**CARBOHYDRATE TRANSPORT AND METABOLISM**					
*gntK*	0.11	<0.0001	0.65 ± 0.24	Chromosome	Gluconate kinase 1
*shf*	0.35	0.0001	0.25 ± 0.09	pCP301	Putative carbohydrate transport protein
*ptsG*	0.32	0.0200	ND	Chromosome	Glucose-specific PTS system IIBC components
*amyA*	0.33	0.0003	0.43 ± 0.04	Chromosome	Cytoplasmic alpha-amylase
*gapC*	0.42	0.0045	ND	Chromosome	Glyceraldehyde-3-phosphate dehydrogenase
*yhcH*	0.43	0.0174	ND	Chromosome	Hypothetical protein
*treA*	0.44	0.0004	ND	Chromosome	Trehalase
*talA*	0.47	0.0001	ND	Chromosome	Transaldolase A
*yhcI*	0.47	0.0196	ND	Chromosome	N-acetylmannosamine kinase
*glk*	0.50	0.0002	ND	Chromosome	Glucokinase
*fucA*	2.19	0.0005	ND	Chromosome	L-Fuculose phosphate aldolase
**VIRULENCE**					
*icsA/virG*	0.33	0.0001	0.24 ± 0.08	pCP301	Intra- and intercellular spread, adhesion
*virK*	0.43	0.0002	0.18 ± 0.05	pCP301	Hypothetical protein
**CELL WALL/MEMBRANE/ENVELOPE BIOGENESIS**					
*rfbU*	0.32	0.0010	0.45 ± 0.12	pCP301	UDP-sugar hydrolase
*mdoB*	0.34	<0.0001	0.22 ± 0.08	Chromosome	Phosphoglycerol transferase I
*slyB*	0.41	0.0025	0.19 ± 0.06	Chromosome	Putative outer membrane protein
*slp*	0.32	0.0006	0.22 ± 0.08	Chromosome	Outer membrane protein induced after carbon starvation
*ecnB*	0.48	0.0007	0.65 ± 0.22	Chromosome	Entericidin B membrane lipoprotein
*pagP*	0.48	0.0120	0.15 ± 0.04	Chromosome	Palmitoyl transferase
*nmpC*	6.35	0.0007	3.15 ± 0.42	Chromosome	Putative outer membrane porin protein C precursor
*msbB2*	0.46	0.0031	0.46 ± 0.08	pCP301	Lipid A biosynthesis
*pmrD*	0.48	0.0166	0.42 ± 0.12	Chromosome	Polymyxin resistance protein B
**ACID RESISTANCE**					
*hdeD*	0.30	0.0078	ND	Chromosome	Acid-resistance membrane protein
*hdeB*	0.31	0.0132	ND	Chromosome	Acid-resistance protein
*hdeA*	0.32	0.0048	0.18 ± 0.03	Chromosome	Acid-resistance protein
*gadA*	0.27	0.0067	0.01 ± 0.003	Chromosome	Glutamate decarboxylase isozyme
*gadB*	0.31	0.0008	ND	Chromosome	Glutamate decarboxylase isozyme
*yhiW*	0.22	<0.0001	0.27 ± 0.06	Chromosome	Putative ARAC-type regulatory protein
*yhiX*	0.24	0.0070	ND	Chromosome	DNA-binding transcriptional regulator GadX
**ENERGY PRODUCTION AND CONVERSION**					
*fadE*	2.31	0.0006	ND	Chromosome	Acyl-CoA dehydrogenase
*gltA*	2.66	0.0143	3.25 ± 0.72	Chromosome	Type II citrate synthase
*tdcD*	3.38	0.0427	ND	Chromosome	Propionate/acetate kinase
*tdcE*	3.46	0.0058	ND	Chromosome	Formate acetyltransferase 3
*hyaC*	0.30	0.0014	ND	Chromosome	Hydrogenase 1 b-type cytochrome subunit
*hyaF*	0.30	0.0015	0.11 ± 0.03	Chromosome	Hydrogenase-1 operon protein hyaF
*hyaB*	0.30	0.0020	0.07 ± 0.01	Chromosome	Hydrogenase 1 large subunit
*appC*	0.32	0.0018	ND	Chromosome	Third cytochrome oxidase, subunit I
*hyaD*	0.32	0.0040	ND	Chromosome	Hydrogenase 1 maturation protease
*hyaA*	0.32	0.0004	ND	Chromosome	Hydrogenase-1 small subunit
*hyaE*	0.33	0.0057	ND	Chromosome	Hydrogenase-1 operon protein hyaE
*appB*	0.36	0.0028	ND	Chromosome	Third cytochrome oxidase, subunit II
*appA*	0.47	0.0016	0.6 ± 0.02	Chromosome	Phosphoanhydride phosphorylase
*fucO*	2.01	0.0025	ND	Chromosome	L-1,2-propanediol oxidoreductase
*sdhD*	2.07	0.0425	ND	Chromosome	Succinate dehydrogenase cytochrome b556 small membrane subunit
*sucC*	2.07	0.0258	ND	Chromosome	Succinyl-CoA synthetase subunit beta
*sdhC*	2.26	0.0288	2.94 ± 0.75	Chromosome	Succinate dehydrogenase cytochrome b556 large membrane subunit
*ykgF*	2.30	0.0241	ND	Chromosome	Hypothetical protein
*ykgE*	2.37	0.0301	3.21 ± 0.72	Chromosome	Putative dehydrogenase subunit
**INORGANIC ION TRANSPORT AND METABOLISM**					
*katE*	0.39	0.0103	0.32 ± 0.05	Chromosome	Hydroperoxidase II
*narU*	0.43	0.0004	ND	Chromosome	Nitrite extrusion protein 2
*bfr*	0.48	0.0001	ND	Chromosome	Bacterioferritin
**LIPID TRANSPORT AND METABOLISM**					
SF2149	0.45	0.0005	ND	Chromosome	Lipid kinase
*ybhO*	0.50	0.0147	ND	Chromosome	Cardiolipin synthase 2
*glpF*	2.33	0.0129	ND	Chromosome	Glycerol diffusion facilitator protein
**POST-TRANSLATIONAL MODIFICATION, PROTEIN TURNOVER, CHAPERONES**					
*ybjX*	0.04	<0.0001	0.58 ± 0.13	Chromosome	Putative enzyme
*cbpA*	0.31	0.0061	ND	Chromosome	Curved DNA-binding protein CbpA
*yccD*	0.44	0.0002	0.32 ± 0.09	Chromosome	Chaperone-modulator protein CbpM
*yeaA*	2.02	0.0026	1.75 ± 0.33	Chromosome	Methionine sulfoxide reductase B
*cysU*	2.08	0.0264	1.64 ± 0.52	Chromosome	Sulfate/thiosulfate transporter subunit
*ipgA*	2.38	0.0003	ND	pCP301	IpgA, similarities to IpgE, putative chaperone
**SECONDARY METABOLITES BIOSYNTHESIS, TRANSPORT AND CATABOLISM**					
*ycaC*	0.43	0.0022	ND	Chromosome	Hypothetical protein
**SIGNAL TRANSDUCTION MECHANISMS**					
*phoP*	0.002	0.0008	0	Chromosome	DNA-binding transcriptional regulator PhoP
*phoQ*	0.002	0.0003	0	Chromosome	Sensor protein PhoQ
*rstA*	0.07	0.0003	0.23 ± 0.05	Chromosome	DNA-binding transcriptional regulator RstA
*cstA*	2.08	0.0256	1.02 + 0.32	Chromosome	Carbon starvation protein
**TRANSCRIPTION**					
*glcC*	2.47	0.0186	3.52 + 1.34	Chromosome	DNA-binding transcriptional regulator GlcC
*yhiE*	0.25	0.0002	ND	Chromosome	Hypothetical protein
*cbl*	0.41	0.0328	ND	Chromosome	Transcriptional regulator Cbl
*adiY*	0.44	0.0016	ND	Chromosome	Putative ARAC-type regulatory protein
*cspH*	2.06	0.0308	ND	Chromosome	Cold shock-like protein
*melR*	2.09	0.0364	2.54 ± 0.83	Chromosome	DNA-binding transcriptional regulator MelR
*hcaR*	2.11	0.0096	ND	Chromosome	DNA-binding transcriptional regulator HcaR
*yjjM*	3.06	0.0004	ND	Chromosome	Hypothetical protein
**TRANSLATION, RIBOSOMAL STRUCTURE AND BIOGENESIS**					
SF4448	0.49	0.0355	ND	Chromosome	tRNA
*yhaR*	3.65	0.0041	5.07 ± 1.21	Chromosome	Hypothetical protein
SF4512	0.50	0.0293	ND	Chromosome	tRNA
**GENERAL FUNCTION PREDICTION ONLY**					
*yoaE*	7.14	<0.0001	3.18 ± 0.83	Chromosome	Putative transport protein
*yjgB*	0.41	0.0004	ND	Chromosome	Putative oxidoreductase
*yjbJ*	0.41	0.0007	ND	Chromosome	Putative stress-response protein
SF1795	0.48	<0.0001	ND	Chromosome	Putative glyceraldehyde-3-phosphate dehydrogenase A
*yciG*	0.49	0.0003	ND	Chromosome	Hypothetical protein
*ykgG*	2.15	0.0080	ND	Chromosome	Putative transporter
SF3152	3.57	0.0110	ND	Chromosome	Putative L-serine deaminase
**FUNCTION UNKNOWN**					
*yrbL*	0.02	<0.0001	0.07 ± 0.01	Chromosome	Hypothetical protein
SF1400	0.04	0.0005	0.21 ± 0.04	Chromosome	Hypothetical protein
*ycgW*	0.09	<0.0001	ND	Chromosome	Hypothetical protein
SF2261	0.17	0.0051	0.04 ± 0.01	Chromosome	Hypothetical protein
SF1401	0.34	0.0034	ND	Chromosome	Hypothetical protein
*ygaM*	0.44	0.0029	ND	Chromosome	Hypothetical protein
*yejG*	2.72	0.0114	3.88 ± 0.92	Chromosome	Hypothetical protein
SF0979	0.30	0.0018	ND	Chromosome	Hydrogenase-1 operon protein
SF1736	0.31	0.0013	ND	Chromosome	Hypothetical protein
*elaB*	0.41	0.0002	ND	Chromosome	Hypothetical protein
*yjiD*	0.41	0.0303	ND	Chromosome	Hypothetical protein
*ybfG*	0.43	0.0054	ND	Chromosome	Hypothetical protein
SF4340	0.43	0.0016	ND	Chromosome	Putative carnitine operon oxidoreductase
SF3143	0.45	0.0002	ND	Chromosome	Hypothetical protein
SF2823	0.47	0.0092	ND	Chromosome	Hypothetical protein
*yqjE*	0.48	0.0065	0.58 ± 0.21	Chromosome	Hypothetical protein
*yqjD*	0.49	0.0031	ND	Chromosome	Hypothetical protein
SF0551	0.50	0.0197	ND	Chromosome	Putative homeobox protein
*ipgB1*	2.03	0.0017	ND	Chromosome	IpgB1, secreted by the Mxi-Spa machinery, function unknown
SF1446	2.09	0.0030	ND	Chromosome	Hypothetical protein
SF0572	2.16	0.0447	ND	Chromosome	Hypothetical protein

a *WT, wild type; ND, not determined*.

b *The differentially expressed genes of microarrays were defined by change ratio> = 2, P < 0.05*.

c *The P-values for the DEGs of microarrays*.

d *qRT-PCR data are given as means ± standard deviations of results from three independent experiments*.

To identify *S. flexneri* PhoP-regulated genes, the online relational databases (http://genolist.pasteur.fr) was used to search the genes with putative PhoP-binding motif in their promoter regions. A PhoP recognition motif was generated based on conserved pattern PhoP box [5′-(T/G) GTTTA-N5-(T/G) GTTTA- 3′] identified in *S. typhimurium* and *E. coli* (Kato et al., 1999; Lejona et al., 2003). The *Sf*301 genome was searched and 38 putative PhoP recognition motifs were detected (Table 5). Among these genes/operons, *phoPQ, mgtA, slyB, rstAB, hdeAB, pagP, yrbL, yoaE, yhiWX*, and *shf* –*rfbU*-*virK*-*msbB2* were reported as members of the PhoPQ regulon in other bacteria (Kato et al., 1999; Lejona et al., 2003; Minagawa et al., 2003; Zwir et al., 2005), while *icsA* and *ipaH7.8* have not been reported as PhoPQ-regulated genes before (Table 5). The transcriptional levels of these 12 genes or operons showed differential expression both in the microarray and qRT-PCR analyses (Table 4).

**Table 5:** Prediction of PhoP-regulated genes in *Sf*301.

**Gene**	**Location**	**Predicted PhoP binding sites**	**Description or predicted function**
*phoP*	Chromosome	t**GGTTTA**tttaa**TGTTTA**c	DNA-binding transcriptional regulator PhoP
*yoaE*	Chromosome	a**TGTTTA**actcc**CGTTTA**a	Transporter
SF1755	Chromosome	c**CGTTTA**aaatt**CGTTTA**g	Porin
*yrbL*	Chromosome	t**TGTTTA**ggttt**TGTTTA**a	Hypothetical protein
*mgtA*	Chromosome	t**GGTTTA**tcgtt**GGTTTA**g	Magnesium-transporting ATPase MgtA
*manX*	Chromosome	t**TAAACG**ggagt**TAAACA**t	PTS system mannose-specific transporter subunits IIAB
*insA*	Chromosome	c**TAAACG**aattt**TAAACG**g	Insertion element IS1 protein InsA
*treR*	Chromosome	c**TAAACC**aacga**TAAACC**a	Trehalose repressor
*mdoB*	Chromosome	t**TAAACG**ttggc**TAAACG**g	Phosphoglycerol transferase I
*uspF*	Chromosome	c**GcTTTA**ggtct**GGTTTA**t	Stress-induced protein
SF1625	Chromosome	a**GGaTTA**aaatt**GGTTTA**a	Hypothetical protein
*sbcD*	Chromosome	a**GaTTTA**tgaca**GaTTTA**t	Exonuclease SbcD
*icsA*	pCP301	t**GGTTgA**ggctt**TGTTTA**a	Hypothetical protein
*yffB*	Chromosome	t**GaTTTA**attct**GGTTaA**a	Reductase
*ygaU*	Chromosome	t**GaTTTA**attct**GGTTaA**a	LysM domain/BON superfamily protein
*ygaC*	Chromosome	a**GGTTcA**tcgcg**GcTTTA**t	Hypothetical protein
SF2987	Chromosome	g**TGTTTA**cctct**GcTTTA**t	Hypothetical protein
*rpsL*	Chromosome	c**GtTTTA**ttacg**TGTTTA**c	30S ribosomal protein S12
*malP*	Chromosome	t**GGTTTg**cacta**GcTTTA**a	Maltodextrin phosphorylase
SF4150	Chromosome	a**GGaTTA**tctgc**GGTTTt**t	Hypothetical protein
*ubiC*	Chromosome	a**GGTTcA**acagc**GtTTTA**c	Chorismate pyruvate lyase
SF1773	Chromosome	g**aGTTTA**atggc**GGTTaA**g	Acetyltransferase
*yjjM*	Chromosome	t**GtTTTA**aatcg**GGTTTt**a	Hypothetical protein
*lpdA*	Chromosome	t**TGTTTA**aaaat**TGTTaA**c	Dihydrolipoamide dehydrogenase
*cbpA*	Chromosome	c**TGTTTA**aaata**TGTTcA**g	Curved DNA-binding protein CbpA
*yajG*	Chromosome	a**GGTTTc**gtcct**GGTTTt**t	Polymerase/proteinase
*ycbK*	Chromosome	t**GcTTTA**cgggc**GGTTaA**g	Hypothetical protein
*ycjY*	Chromosome	a**GGTcTA**atcat**GaTTTA**g	Hypothetical protein
*sdaA*	Chromosome	c**GGTTTt**tgatt**aGTTTA**a	L-serine deaminase
SF1507.1	Chromosome	t**GaTTTA**ttaga**GcTTTA**t	Transmembrane anchor protein
*slyB*	Chromosome	t**tGTTTA**taatt**GGTTgA**t	Hypothetical protein
*ybiC*	Chromosome	a**TGgTTA**actcc**TGTTTA**t	Hypothetical protein
*mipA*	Chromosome	t**TGTTTA**aggaa**TGaTTA**a	structural protein MipA
*shf*	pCP301	t**TGTTTA**tgaat**TGTTgA**t	Carbohydrate transport protein
*dppA*	Chromosome	t**TtTTTA**atctt**TGTTTg**t	Dipeptide transport protein
*hdeA*	Chromosome	c**TGTaTA**tgtca**TGTTgA**t	Acid stress chaperone HdeA
*yhiW*	Chromosome	a**TGTTTg**ggcga**TtTTTA**t	Putative ARAC-type regulatory protein
*ipa*H7.8	pCP301	a**TGTgTA**tcgtt**TtTTTA**c	Invasion plasmid antigen

*The genes with a putative PhoP-binding motif in Sf301 were searched based on the PhoP box pattern [5′-(T/G) GTTTA-N5-(T/G) GTTTA-3′]. The putative PhoP binding sites in the promoter region were restricted to 400 bp before the start codon with at most 2 nt not matching. The bold sequences represent the PhoP box pattern in the predicted PhoP binding sites*.

**Figure 6:**
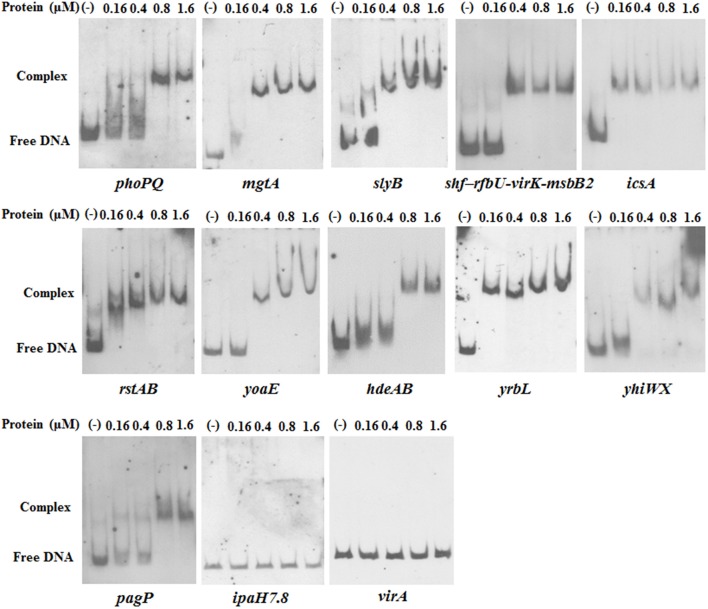
EMSA analysis of PhoP with the putative promoter regions. His-tagged PhoP was purified and phosphorylated (PhoP-P) by incubation with 50 mM acetylphosphate. The putative promoter regions of the genes/operons *phoPQ, mgtA, slyB, shf*–*rfbU*-*virK*-*msbB2, icsA, rstAB, yoaE, hdeAB, yrbL, yhiWX, pagP*, and *ipaH*7.8 were PCR-amplified. DNA probes were purified and labeled with digoxigenin. Gel shift reactions were performed by incubating the labeled probe with increasing concentrations of PhoP-P (range, 0.16–1.6 μM). Lane 1 of each blot contained a no-protein control. All samples were electrophoresed on a non-denaturing polyacrylamide gel and blotted onto a nylon membrane. After incubation with anti-digoxigenin antibody, CSPD chemiluminescent reagent was added. The DNA fragment within the *virA* coding region was used as a negative control.

To verify those 12 predicted PhoP target genes/operons above in *Shigella*, EMSAs were performed. The recombinant PhoP-P resulted in a mobility shift of the fragments upstream of 11 genes/operons (*phoPQ, mgtA, slyB, icsA, shf* –*rfbU*-*virK*-*msbB2, rstAB, yoaE, hdeAB, yrbL, yhiWX*, and *pagP*) in a concentration-dependent manner, but did not bind to the fragment upstream of *ipaH*7.8 (Figure 6). As a negative control, the DNA fragment of *virA* coding sequence (without PhoP box motif) did not form a complex with PhoP under the same conditions (Figure 6).

**Figure 7:**
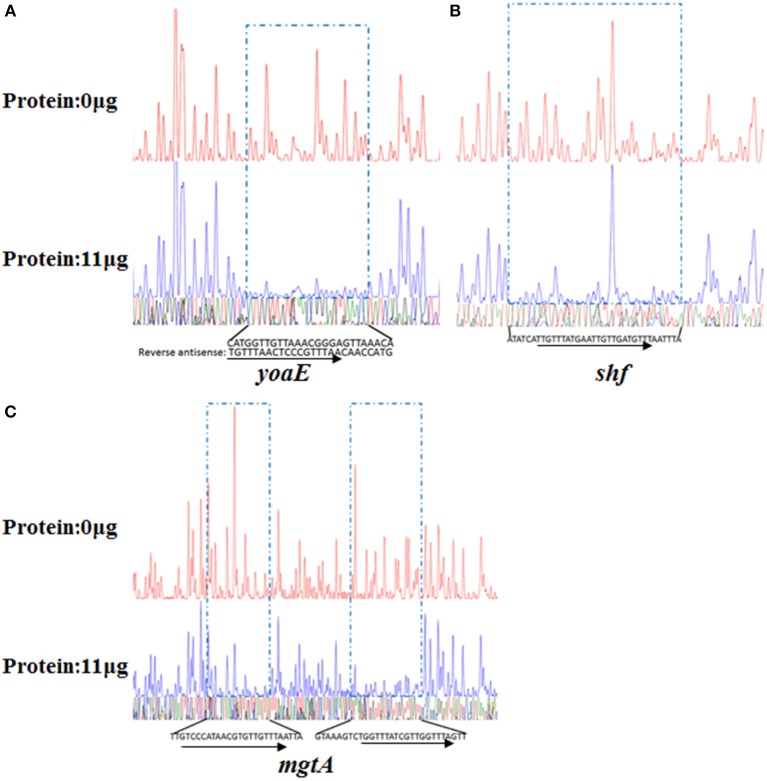
Identification of the PhoP-protected cis-elements in the promoter regions of *mgtA, yoaE*, and *shf* using DNase I footprinting assay. Probes used were labeled with FAM dye and were illustrated in Material and Methods. Different amounts of PhoP protein were used for the assay. The fmol DNA Cycle Sequencing System was used for DNA sequencing reactions and the four sequencing results (G, A, T, and C) were marked with four different colors separately and then merged together. The electropherograms were aligned together with the usage of GeneScan-LIZ500. The areas protected of yoaE **(A)**, shf **(B)**, and mgtA **(C)** from DNase I by PhoP were sequenced, and inside the sequence, bases consistent with the PhoP box motif are arrowed.

The PhoP-P binding motif sequences in the promoters of *yoaE, shf* and *mgtA* were identified by DNase I footprinting assay. A 25-nt protected sequence in the promoter region of *yoaE* (−107 to −83 bp) was identified (Figure 7A), and a 35-nt protected sequence was located at −115 to −81 bp in the upstream of the translational start site of *shf* (Figure 7B). There exist two protected sequences in the *mgtA* promoter (−184 to −159 bp and −152 to −124 bp) (Figure 7C). All protected sequences were in accordance with PhoP binding consensus motif (Figure 7, Table 5).

**Figure 8:**
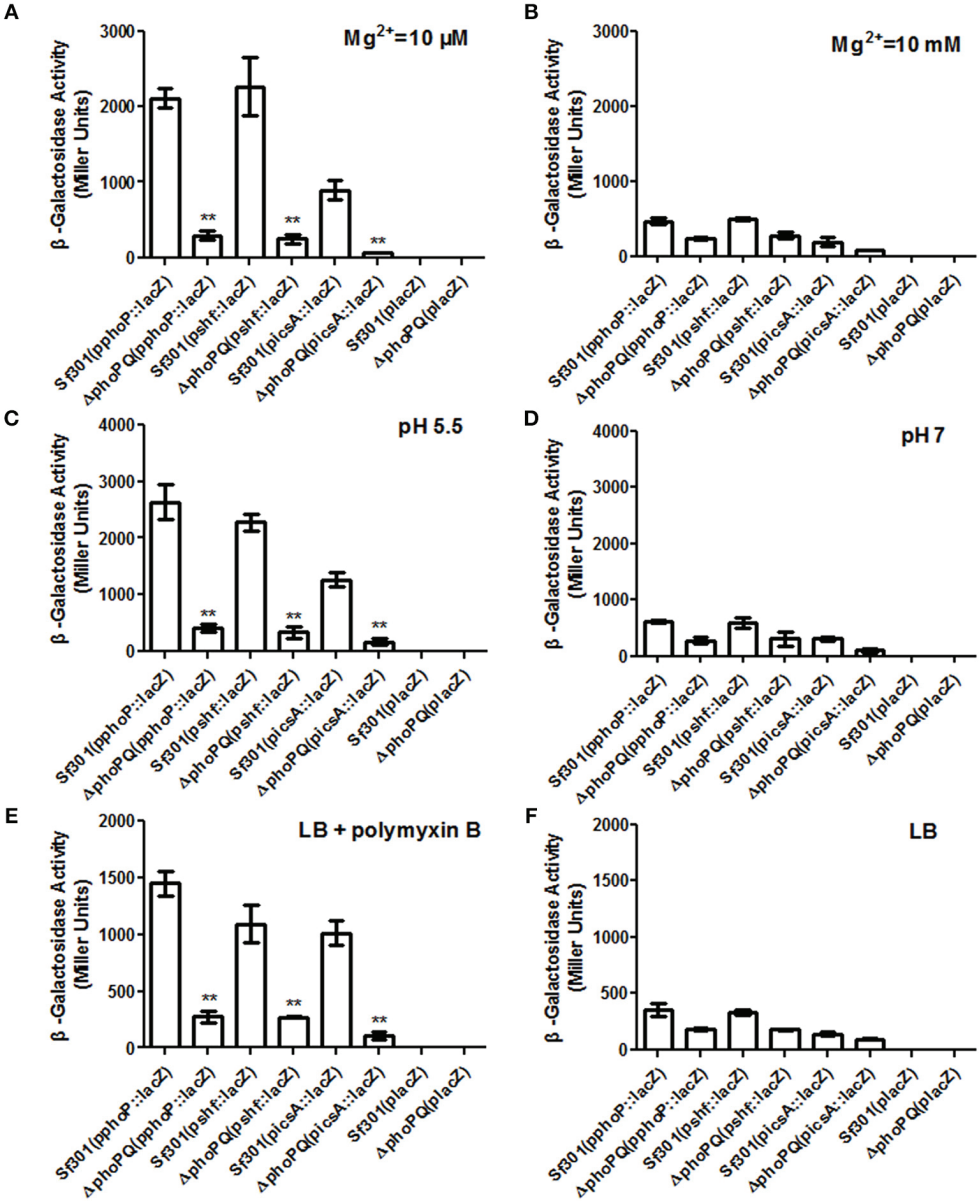
β-Galactosidase activity of *phoP, shf*, and *icsA* LacZ reporter strains in conditions of low Mg^2+^, acidic pH, or presence of polymyxin B. β-Galactosidase activity from the p*phoP*::*lacZ*, p*shf* ::*lacZ* and p*icsA*::*lacZ* transcriptional fusion in *Sf*301 and Δ*phoPQ* were determined. Bacteria were grown for 8 h in N medium with 10 μM MgCl_2_ for low Mg^2+^ **(A)** or N medium with 10 mM MgCl_2_ for high Mg^2+^ **(B)**. Bacteria were grown for 8 h in E glucose broth at pH 5.5 **(C)** or pH 7 **(D)**. Bacteria were grown for 6 h in LB then treated with 25 μg/ml polymyxin B **(E)** or without polymyxin B **(F)** for 1 h. The *Sf*301(p*lacZ*) and Δ*phoPQ*(p*lacZ*) were used as empty plasmid controls. The data correspond to mean values from three independent experiments performed in each case. Error bars correspond to standard deviations. ^**^P < 0.01.

Furthermore, the transcriptional levels of genes with PhoP binding activities in low Mg^2+^, acidic pH, or presence of polymyxin B were detected by qRT-PCR. Among those genes, transcriptional levels of *phoP, shf* and *icsA* were up-regulated significantly in all three environmental stress conditions (Tables S6–S8). Three LacZ reporter plasmids with the promoter regions of the genes (p*phoP*::*lacZ*, p*shf* ::*lacZ*, and p*icsA*::*lacZ*) were then constructed to confirm PhoPQ regulation on those genes expression. After transformation of the plasmids into Δ*phoPQ* or *Sf*301 and culture in different mediums, β-galactosidase activity was detected. In low Mg^2+^ (10 μM) medium, the expression of *phoP, shf*, and *icsA* in *Sf*301 was 7.2, 9, and 12.9-fold higher, respectively, than that in Δ*phoPQ* (Figure 8A), while in high Mg^2+^ (10 mM) medium, the expression of *phoP, shf*, and *icsA* in *Sf*301 was only 1.9, 1.8, and 2.2-fold higher, respectively, than that in Δ*phoPQ* (Figure 8B). Under acidic pH (pH 5.5) conditions, the expression of *phoP, shf*, and *icsA* in *Sf*301 was 6.4, 6.6, and 7.1-fold higher, respectively, than that in Δ*phoPQ* (Figure 8C). In contrast, at pH 7, the expression of *phoP, shf*, and *icsA* in *Sf*301 was only 2.2, 2, and 3-fold higher, respectively, than that in Δ*phoPQ* (Figure 8D). In the presence of polymyxin B (25 μg/ml) in LB medium, the expression of *phoP, shf* and *icsA* in *Sf*301 was 5.2, 4, and 9.2-fold higher, respectively, than that in Δ*phoPQ* (Figure 8E), while in LB medium only, the expression of *phoP, shf* and *icsA* in *Sf*301 was only 2, 1.9, and 1.5-fold higher, respectively, than that in Δ*phoPQ* (Figure 8F). It suggested that the expressions of *phoP, shf* and *icsA* were regulated by PhoPQ.

### Validation of IcsA regulation by PhoPQ in *Shigella* virulence

**Figure 9:**
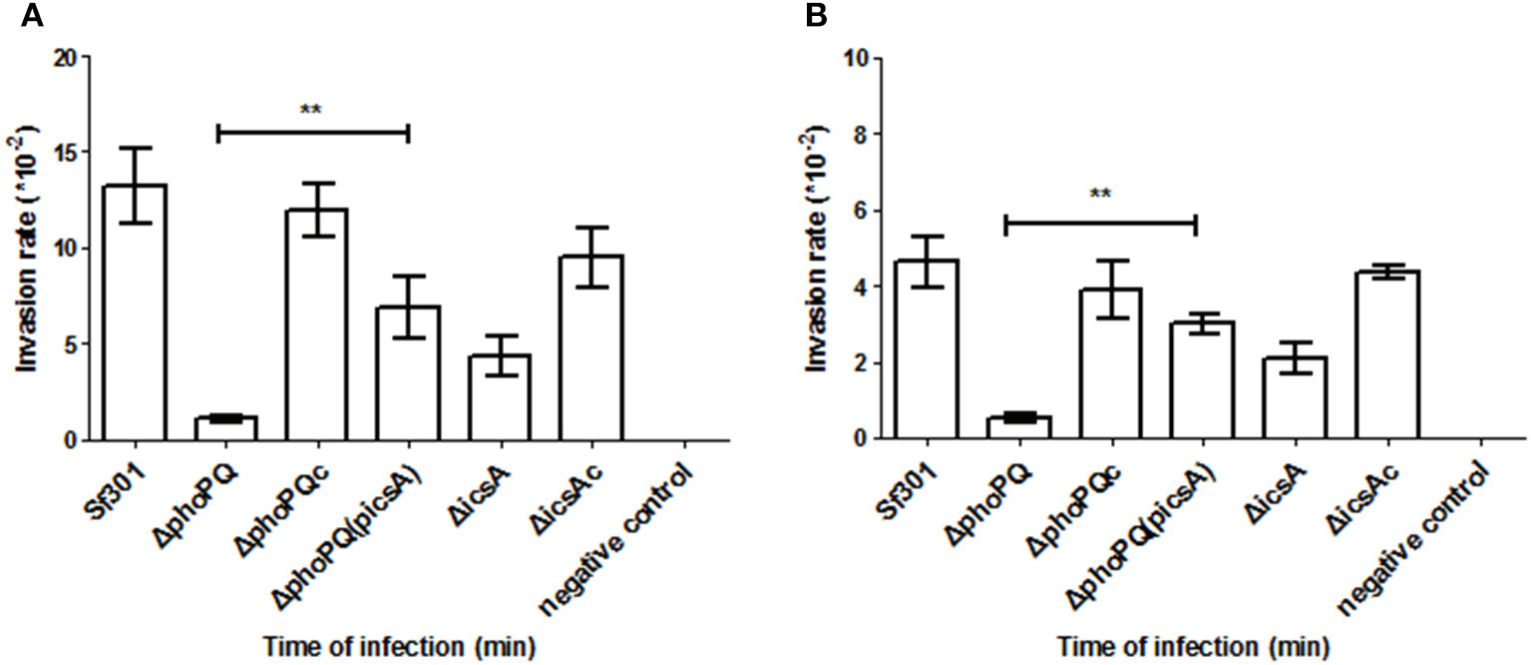
Invasion ability of Δ*phoPQ(picsA)* and Δ*icsA* into HeLa cells and Caco-2 cells. The gentamicin protection assay was used as a cellular model to evaluate the effect of Δ*phoPQ* complemented with *icsA* on the virulence of *Shigella*. Bacteria grown to logarithmic phase were added to the cells for 30 min. Gentamicin was then added to the medium to kill extracellular bacteria. Colonies of lysates on LB plates were counted. The invasion rate refers to the number of intracellular bacteria divided by the number of inoculated bacteria and multiplied by 10,000. **(A)** Bacterial ability to invade HeLa cells. **(B)** Bacterial ability to invade Caco-2 cells. Values are means ± standard deviations from three independent wells. ^**^P < 0.01.

As virulence is the key factor in *Shigella* pathogenesis, we focused on searching for PhoP target genes that are associated with *Shigella* virulence. Four PhoP-regulated genes (*rstA, icsA, yrbL*, and *yoaE*) that may be involved in *Shigella* virulence were deleted from *Sf*301, respectively. The virulence of these mutant strains was evaluated by the gentamicin protection assay on HeLa cells and only Δ*icsA* decreased virulence in HeLa cells compared to *Sf*301 (Figure S2). IcsA is one of the virulence factors required for *Shigella* pathogenesis (Bernardini et al., 1989; Brotcke Zumsteg et al., 2014), and its expression is regulated by PhoPQ based on results of the microarray, qRT-PCR, EMSA and β-galactosidase activity assay in our study. The transcriptional level of *icsA* in Δ*phoPQ* was significantly reduced both in the microarray (3-fold down-regulated) and qRT-PCR (4.2-fold down-regulated) compared to that of *Sf*301 (Table 4). A highly conserved motif is found in the promoter region of *icsA* and PhoP-P results in a mobility shift of the fragments upstream of *icsA* (Table 5, Figure 6). The β-galactosidase activities of *icsA* in *Sf*301 were significantly higher than that in Δ*phoPQ* (12.9, 7.1, and 9.2-fold higher, respectively) in the environments of low Mg^2+^, acidic pH or presence of polymyxin B (Figures 8A,C,E). As the *phoPQ* knockout diminished *S. flexneri* virulence, an *icsA* expression plasmid (p*icsA*) was introduced into the Δ*phoPQ* strain [Δ*phoPQ*(p*icsA*)] to observe whether virulence of Δ*phoPQ* could recover. A *Shigella icsA* deletion mutant strain (Δ*icsA*) and its complementation strain (Δ*icsAc*) served as controls. The invasion rate of HeLa cells or Caco-2 cells by Δ*phoPQ*(p*icsA*) was 6.4 and 5.7-fold higher than that of Δ*phoPQ*, respectively (Figures 9A,B). Δ*phoPQ*(p*icsA*) resulted in more membrane ruffles indicative of actin cytoskeleton changes in HeLa cells compared to Δ*phoPQ* (Figure 2F). In the guinea pig Sereny test, guinea pigs inoculated with Δ*phoPQ(*p*icsA)* showed restored virulence (24 h + or ++ and 48 h ++ or +++) compared to Δ*phoPQ* (24 h– and 48 h– or +) (Figure 3, Table 3). Guinea pig eyes infected with Δ*phoPQ(*p*icsA)* showed markedly more inflammatory reactions including epithelial desquamation and neutrophil infiltration in the pathological examination compared to Δ*phoPQ*-infected eyes (Figure 4I). As a control, the virulence of Δ*icsA* was decreased both in the gentamicin protection assay (2.7 and 2.2-fold lower, Figures 9A,B) and guinea pig Sereny test (24 h– or + and 48 h +, Figure 3, Table 3), compared to *Sf*301. It indicated that the virulence of Δ*phoPQ*(p*icsA*) could be restored partly by complementation with p*icsA*, but it still did not reach the level of *Sf*301.

## Discussion

The PhoPQ TCS is widely involved in the regulation of virulence in a variety of pathogenic bacteria, including *Salmonella, Yersinia, Neisseria, Mycobacterium, Erwinia, Pseudomonas*, and *Serratia*. The deletion of *phoPQ* in these organisms have shown a significantly decrease in virulence (Miller et al., 1989; Flego et al., 2000; Oyston et al., 2000; Johnson et al., 2001; Perez et al., 2001; Gooderham et al., 2009; Bozue et al., 2011; Barchiesi et al., 2012). Moss's previous works have shown that a *phoP* mutant decreased the inflammatory response and was more sensitive to PMNs in *S. flexneri* (Moss et al., 2000), which indicates that PhoPQ has the function of virulence regulation in *Shigella*. In the present study, we demonstrate that the PhoPQ system regulates the virulence of *Shigella* both *in vivo* and *in vitro*. In the HeLa cell and Caco-2 cell invasion models, the invasion ability of Δ*phoPQ* declined significantly compared with that of *Sf*301 (Figure 1) and no obvious membrane ruffling was observed in Δ*phoPQ* infection cells (Figure 2). In the guinea pig keratoconjunctivitis model, guinea pigs infected with Δ*phoPQ* displayed a slight conjunctival inflammation (Figure 3, Table 3) and fewer pathologic changes in the pathological examination (Figure 4).

Extracellular Mg^2+^, pH and antimicrobial peptides have been reported as input signals of the PhoPQ system and these signals can regulate the expression of PhoP in *Salmonella* and other bacteria (Garcia Vescovi et al., 1996; Gunn and Miller, 1996; Bearson et al., 1998; Lejona et al., 2003; Barchiesi et al., 2012; Shprung et al., 2012). Polymyxin B is an important antimicrobial agent extensively used clinically for the effective treatment of multi-drug resistant Gram-negative infections (Bergen et al., 2015; Brown and Dawson, 2017). As the *Shigella phoPQ* shares high similarity with that of *Salmonella* (Tables S2, S3), we predict that the *Shigella* PhoPQ also functions in responding the signals of extracellular Mg^2+^, pH and antimicrobial peptides. In the present study, we demonstrate that the PhoPQ system allows *Shigella* to tolerate scarce environmental Mg^2+^ availability, acidic pH, and high concentrations of polymyxin B. The Δ*phoPQ* showed growth deficiency in low Mg^2+^ or acidic pH conditions compared with *Sf*301 (Figures 5B,D). The survival rates of Δ*phoPQ* were significantly lower than those of *Sf*301 in the presence of polymyxin B (Figure 5E). We also demonstrate that the expression of PhoPQ is promoted under those three environmental stress conditions (low Mg^2+^, acidic pH or presence of polymyxin B) (Tables S6–S8).

Though the PhoPQ system shares similar functions in Gram-negative bacteria, the regulons of PhoPQ are diverse in different bacteria (Groisman, 2001). In the present study, we have screened the PhoPQ-regulated genes in *Shigella*. Firstly, DNA microarray was performed to compare the transcriptional profiles of *Sf*301 and Δ*phoPQ*, and 117 DEGs were found. The function of these genes were involved in metal ion transport (*katE, narU, bfr*), acid resistance (*hdeABCD, gadAB, yhiWX, xasA*), LPS modification and antibacterial peptide tolerance (*rfbU, mdoB, slyB, pagP, msbB2, pmrD*), signal transduction (*phoPQ, rstA, cstA*), bacterial virulence (*icsA, virK*), respiratory and energy metabolism (*hyaABCDEF, appABC*) (Table 4).

**Figure 10:**
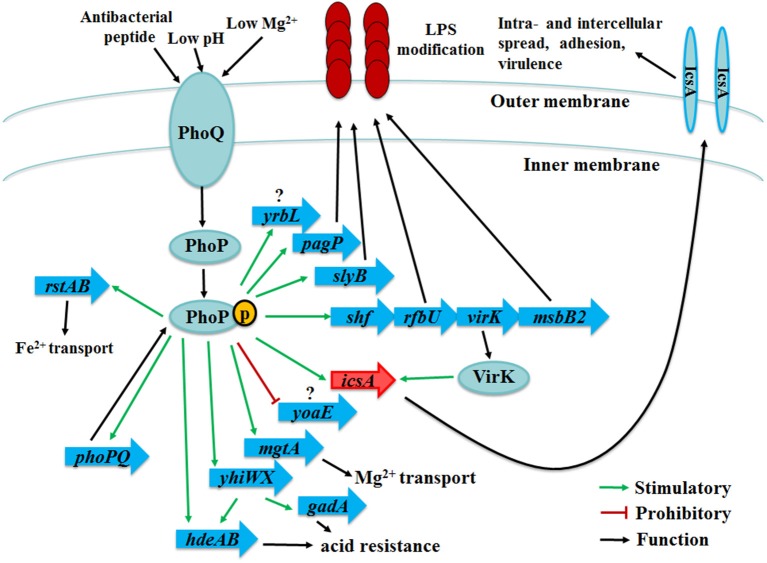
Proposed regulation model of PhoPQ in *Shigella*. In this work, we have determined 11 genes or operons regulated by PhoPQ. Among these genes/operons, *mgtA* is involved in Mg^2+^ transport. *pagP, slyB, rfbU* and *msbB2* act on LPS modification and antibacterial peptide tolerance. *hdeAB* and *yhiWX* function in acid resistance. *icsA* is the first time discovered to be regulated by PhoPQ and is involved in the cell-to-cell spreading process and bacterial virulence. *virK* is an essential virulence determinant involved in the expression of the *icsA* gene at the post-transcriptional level. *rstAB* is another two-component system that senses the environmental pH and regulates Fe^2+^ transport*. phoPQ* is autoregulated by PhoP. The functions of *yrbL* and *yoaE* are still unknown. Green arrows represent positive regulatory pathways. Red arrows represent negative regulatory pathways. Black arrows represent functions of PhoP-regulated genes.

The promoter of PhoP-regulated genes contains a PhoP recognition motif [(T/G)GTTTA-5nt-(T/G)GTTTA] that has been termed the PhoP box in *S. typhimurium* and *E. coli* (Kato et al., 1999; Lejona et al., 2003). Considering the high conservation of *phoPQ* genes (Tables S2, S3), we screened 38 suspected PhoP target operons in *Sf*301 genome based on the PhoP box motif using the online relational databases (http://genolist.pasteur.fr). The putative PhoP-regulated genes were verified by EMSA (Figure 6). Eleven PhoP-regulated genes or operons were found. The *phoPQ* operon demonstrated autoregulation in *Shigella* (Figure 8). MgtA is involved in magnesium transport (Smith et al., 1998; Gall et al., 2016). PagP (Pilione et al., 2004; Bishop, 2005), SlyB (Plesa et al., 2006), RfbU (Yao and Valvano, 1994) and MsbB2 (Somerville et al., 1999; D'Hauteville et al., 2002) act on LPS modification and antibacterial peptide tolerance. HdeAB (Gajiwala and Burley, 2000) and YhiWX (Ma et al., 2002) function in acid resistance. RstAB is another two-component system that senses environmental pH and is required for the virulence of pathogenic *E. coli* (Cabeza et al., 2007; Jeon et al., 2008; Gao et al., 2015). IcsA is the first time discovered to be regulated by PhoPQ in our study and is involved in the cell-to-cell spreading process and bacterial virulence (Bernardini et al., 1989; Goldberg and Theriot, 1995; Brotcke Zumsteg et al., 2014). VirK is an essential virulence determinant involved in the expression of the gene *icsA* at the post-transcriptional level (Nakata et al., 1992; Detweiler et al., 2003). The functions of YrbL and YoaE are unknown (Figure 10). Through the DNase I footprinting assay, we demonstrated the *Shigella* PhoP binding sequences fit the PhoP box motif (Figure 7).

To search for PhoP target genes that are associated with *Shigella* virulence, four genes that may be involved in virulence (*rstA, icsA, yrbL*, and *yoaE*) were deleted from *Sf*301, respectively. The virulence of those mutant strains was evaluated using the gentamicin protection assay on HeLa cells, and deletion of *icsA* decreased *Shigella* virulence (Figure S2). IcsA is a virulence factor involved in the cell-to-cell spreading process and required for *Shigella* pathogenesis (Bernardini et al., 1989; Ogawa et al., 2005). In the present study, we have demonstrated *icsA* is a positively PhoP-regulated gene and PhoPQ regulates *S. flexneri* virulence in an *icsA*-dependent manner. The transcriptional level of *icsA* in Δ*phoPQ* decreased significantly both in the microarray and qRT-PCR. The PhoP box motif was found in the promoter region of *icsA* and PhoP-P resulted in a mobility shift of the fragments upstream of *icsA* (Table 5, Figure 6). The promoter activities of *icsA* in *Sf*301 were significantly higher than that in Δ*phoPQ* in the environments of low Mg^2+^, acidic pH or presence of polymyxin B (Figures 8A,C,E). We introduced the *icsA* expression plasmid p*icsA* into Δ*phoPQ* and found that the virulence of the Δ*phoPQ*(p*icsA*) strain could be restored partly (Figures 2–4, 9, Table 3). Since the down-regulated level of virulence in Δ*icsA* is not as low as that in Δ*phoPQ* (Figures 2–4, 9, Table 3), we hypothesize *icsA* is not the only PhoP-regulated virulence factor. Besides *icsA*, the *shf-rfbU-virK-msbB2* operon could be another virulence factor regulated by PhoP. This operon is demonstrated as being regulated by PhoP in this study and previous reports (Zwir et al., 2005). MsbB2 acts by catalyzing lipid A acylation (D'Hauteville et al., 2002; Goldman et al., 2008) and RfbU functions in the synthesis of O-antigen (Yao and Valvano, 1994). These two proteins are important in the synthesis of LPS, which is responsible for inflammation of the host. VirK is a cytoplasmic polypeptide required for the bacteria to spread into host cells by being involved in the full expression of the IcsA protein (Nakata et al., 1992; Detweiler et al., 2003).

In summary, we found that the two-component signal transduction system PhoP/PhoQ is involved in the regulation of *S. flexneri* virulence and ability to tolerate low environmental Mg^2+^, acidic pH, and antimicrobial peptide polymyxin B. We identified 117 DEGs, which were involved in Mg^2+^ transport, acid resistance, LPS modification, adhesion and invasion, respiratory and energy metabolism by comparing the transcriptional profiles of Δ*phoPQ* and *Sf*301. We screened out 38 potential PhoP target operons in *S. flexneri* by a bioinformatics search approach and 11 of them were identified to be PhoP-regulated genes/operons by EMSA assays and β-galactosidase assays. One of these genes, *icsA* (a well-known virulence factor), was the first time discovered to be regulated by PhoP. It indicates that the PhoPQ system modulates *S. flexneri* virulence in an *icsA*-dependent manner.

## Author contributions

DQ, XC, and ZLi designed the study; ZLi, XC, MC, LY, YW, XW, ZLv, and YS completed all the experiments. ZLi performed the statistically analysis and made the figures; ZLi, DQ, and XC wrote and revised the manuscript.

### Conflict of interest statement

The authors declare that the research was conducted in the absence of any commercial or financial relationships that could be construed as a potential conflict of interest.

## Supplementary material

Figure S1 The *phoPQ* gene disruption strategy. H1 and H2 refer to the homology regions of *phoPQ*, P1 and P2 refer to the primers of *kan* resistance gene.

Figure S2 The invasion ability of Δ*icsA*, Δ*yoaE*, Δ*yrbL*, and Δ*rstA* to HeLa cells. The bacteria that grew to logarithmic phase were added into the cells for 30 min infection. Then gentamicin was added into the medium to kill extracellular bacteria. Colonies of lysates on LB plates were counted. The invasion rate refered to the number of intracellular bacteria divided by that of inoculated bacteria and multiplied by 10,000. Values are means ± standard deviations from 3 independent wells. ^**^*P* < 0.01.

Table S1 Primers used for qRT-PCR verification of differentially expressed genes in Δ*phoPQ*.

Table S2 Homological analysis of *Sf*301 *phoP/phoQ* with other homologues.

Table S3 Homological analysis of *Sf*301 PhoP/PhoQ with other homologues.

Table S4 Primers used for construction of Δ*icsA*, Δ*yoaE*, Δ*yrbL*, Δ*rstA* and their complementation strain.

Table S5 Differentially expressed genes of Δ*phoPQ* compared to *Sf*301 by microarray and qRT-PCR at early-stationary phase.

Table S6 The transcriptional levels of *phoPQ* and its regulated genes in different concentrations of Mg^2+^ condition.

Table S7 The transcriptional levels of *phoPQ* and its regulated genes in different pH condition.

Table S8 The transcriptional levels of *phoPQ* and its regulated genes with or without polymyxin B condition.
