# Supplementary figures and images for: Virulence and Stress Responses of Shigella flexneri Regulated by PhoP/PhoQ
Source: Front Microbiol. 2018 Jan 15;8:2689. doi: 10.3389/fmicb.2017.02689 (PMC5775216; doi:10.3389/fmicb.2017.02689)

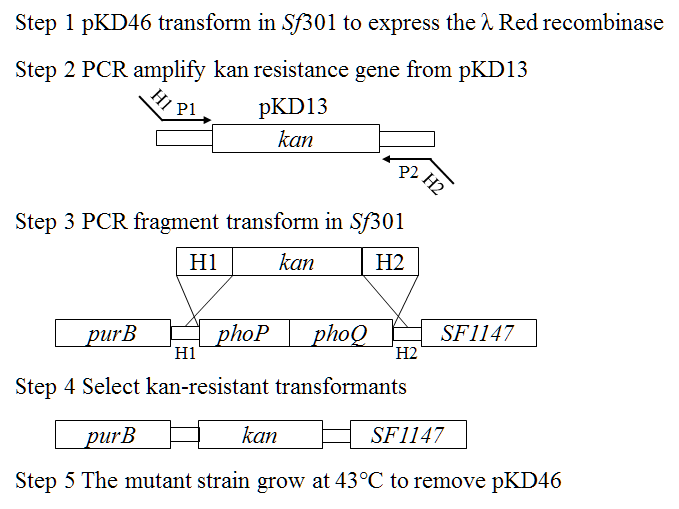

Supplement: Figure S1 — The phoPQ gene disruption strategy. H1 and H2 refer to the homology regions of phoPQ, P1 and P2 refer to the primers of kan resistance gene. [file Image1.TIF]

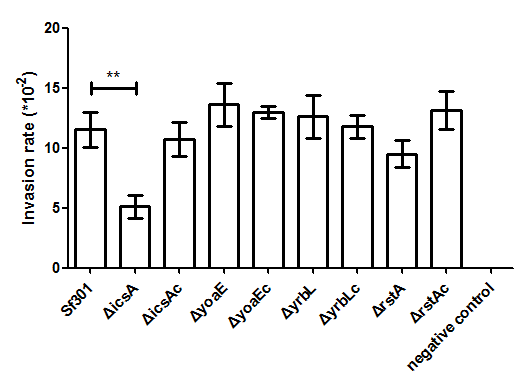

Supplement: Figure S2 — The invasion ability of ΔicsA, ΔyoaE, ΔyrbL, and ΔrstA to HeLa cells. The bacteria that grew to logarithmic phase were added into the cells for 30 min infection. Then gentamicin was added into the medium to kill extracellular bacteria. Colonies of lysates on LB plates were counted. The invasion rate refered to the number of intracellular bacteria divided by that of inoculated bacteria and multiplied by 10,000. Values are means ± standard deviations from 3 independent wells. **P < 0.01. [file Image2.TIF]
