# Supplementary material for: Virulence and Stress Responses of Shigella flexneri Regulated by PhoP/PhoQ
Source: Front Microbiol. 2018 Jan 15;8:2689. doi: 10.3389/fmicb.2017.02689 (PMC5775216; doi:10.3389/fmicb.2017.02689)
Supplement: Table S1 — Primers used for qRT-PCR verification of differentially expressed genes in ΔphoPQ. [file Table1.DOCX]

**TABLE S1****︱Primers used for qRT-PCR verification of differentially expressed genes in *△phoPQ***

| **Primers^a^** | **Sequences（5’-3’）** | **Location^b^** | **Product Length (bp)** |
| --- | --- | --- | --- |
| *rsmC*-F | CTGCCCCGGCGGTAGAAGC | 4573886-4573868 | 289 |
| *rsmC*-R | ACGGCGGGTTGGAGATGAT | 4573761-4573779 |  |
| *phoP*-F | TCGCCGTGAATTATCTAT | 1191221-1191204 | 121 |
| *phoP*-R | TTGTAGCATTAACGAATCTT | 1191101-1191120 |  |
| *phoQ*-F | CATTGTGGTGGTGGATAC | 1190439-1190422 | 99 |
| *phoQ*-R | ATCACTAACAGCAGATTGG | 1190341-1190359 |  |
| *icsA*-F | TCAATCCGTTACTCACTACT | 151013-151032 | 105 |
| *icsA*-R | TGTCGCCATCTGTATCAT | 151100-151117 |  |
| *slyB*-F | AATGGTAGGTCTGTCTCT | 1698004-1698021 | 87 |
| *slyB*-R | TGTACTTGTTTCGCTTCA | 1698090-1698073 |  |
| *yoaE*-F | GGCGATGTGATTGATGTA | 1450151-1450168 | 82 |
| *yoaE*-R | TAACAATGCGAACCAGAT | 1450232-1450215 |  |
| *yrbL*-F | AAGACTGGAGTGGAATAC | 3340791-3340808 | 77 |
| *yrbL*-R | AATCACATCGTAGACATAAC | 3340867-3340848 |  |
| *shf*-F | CATACGCATACTCATACA | 192174-192191 | 175 |
| *shf*-R | CGACCTGAATATAATCCTT | 192348-192330 |  |
| *gntK*-F | CAATAAAGTGTCGCTGAT | 3550003-3549986 | 80 |
| *gntK*-R | AGAGATTCGGATTACCTT | 3549924-3549941 |  |
| *yhiW*-F | CTCATTCGTCGTTCATTCG | 3698513-3698531 | 75 |
| *yhiW*-R | CAGCAGCAGGATACTCTC | 3698587-3698570 |  |
| *gadA*-F | TGATGAATTATATCTTGATGG | 1770402-1770422 | 137 |
| *gadA*-R | ATTGCGGATATTCTTCTT | 1770538-1770521 |  |
| *mdoB*-F | ATAACTATCTCGGACTTG | 4564395-4594378 | 136 |
| *mdoB*-R | TCTTTCATCTCTTTAGGG | 4564260-4564277 |  |
| *xasA*-F | CTCACATTACTCGGATTC | 1771854-1771871 | 136 |
| *xasA*-R | AATAGCAGGAAGAAGACT | 1771960-1771943 |  |
| *appA*-F | GCTTAATTTCCCGCAATC | 1029615-1029632 | 89 |
| *appA*-R | TGAGTTCCGATGGTAATG | 1029703-1029686 |  |
| *yqjE*-F | TTGGTCTTATGAGCCTGATG | 3241833-3241852 | 75 |
| *yqjE*-R | TGGCAATCATCGCATTCA | 3241907-3241890 |  |
| *pagP*-F | CTAACGCAGATGAGAGGAT | 693892-693874 | 97 |
| *pagP*-R | CAGGTGATGGCAGGAATA | 693796-693813 |  |
| *sdhC*-F | TATTCGCCACATGATGAT | 601613-601596 | 96 |
| *sdhC*-R | AGCACGACAGTAATAACA | 601518-601535 |  |
| *ybjX*-F | ATTCTGGCATCGCTGTAG | 870293-870276 | 81 |
| *ybjX*-R | TTCATCCACTCAACGCTTA | 870213-870231 |  |
| *tdcC*-F | GATTACACTGGAATATGC | 3255587-3255570 | 119 |
| *tdcC*-R | TATAACCGAACTTCAGAA | 3255469-3255486 |  |
| *rfbU*-F | TTATTGCTCAGGTAGGAATG | 193113-193132 | 107 |
| *rfbU*-R | CACAATCAACCAACAGAAC | 193219-193201 |  |
| *virK*-F | ATCGGTATCATTAGTCCAA | 193988-194006 | 98 |
| *virK*-R | ACAACATCAACTCTCCTT | 194085-194068 |  |
| *msbB2*-F | GTGATTGATTATGTCTGGAA | 195157-195176 | 93 |
| *msbB2*-R | TACTGACTGAATAAATGGTTT | 195249-195229 |  |
| *pmrD*-F | CAATGGAATGGCTGGCACAA | 2386269-2386250 | 75 |
| *pmrD*-R | AGCATCGCAGAGCATAAGTACA | 2386195-2386216 |  |
| *nanA*-F | CGCCGTTCTACTATCCTT | 3359353-3359336 | 75 |
| *nanA*-R | AACCATCCGCTGAATCAA | 3359279-3359296 |  |
| *slp*-F | CAGTGAACTATCGTAATC | 3631328-3631345 | 158 |
| *slp*-R | CAGGTTATAGGTGGTATT | 3631485-3631468 |  |
| *melR*-F | CGATGGCGATGTGGAATA | 4267086-4267103 | 96 |
| *melR*-R | GTTAGTTGGTGCGGTGTA | 4267181-4267164 |  |
| *ecnB*-F | TGGTGCTTTCAACAGTATTAAC | 4480976-4480997 | 75 |
| *ecnB*-R | CGTTACCGCCATCAGAAA | 4481050-4481033 |  |
| *dppA*-F | ATACGCTGGTCTTCTCTAT | 3678049-3678031 | 76 |
| *dppA*-R | ATCACCTGGCATTCATTC | 3677974-3677991 |  |
| *ibpB*-F | AAGCGACGATAACCACTA | 3885997-3886014 | 79 |
| *ibpB*-R | GCCTTCCAGTTGAATCTC | 3886075-3886058 |  |
| *ybjZ*-F | GGCGGATATTCGTTCTATT | 872562-872580 | 80 |
| *ybjZ*-R | GATATTGCGGATCGTCAT | 872641-872624 |  |
| *rplS*-F | GAGCAACATTATTAAGCAACT | 2743179-2743159 | 75 |
| *rplS*-R | TTCACTTCCACGGTATCA | 2743096-2743113 |  |
| *yejG*-F | ATGGTCGGTGATGTATAAACT | 2301278-2301258 | 76 |
| *yejG*-R | ACCTTCACACTCCAACAC | 2301203-2301220 |  |
| *cysU*-F | TTACAGGAGTTTGATTAC | 2539107-2539090 | 90 |
| *cysU*-R | CAGAGTGTTAATTGAGAA | 2539018-2539035 |  |
| *glcC*-F | TCAGGCTTCTCACAATCA | 3116749-3116766 | 87 |
| *glcC*-R | TTATTTACGCTGGCAAACA | 3116835-3116817 |  |
| *yhaR*-F | CATCAAGATGACCGTGTT | 3251403-3251386 | 76 |
| *yhaR*-R | ATCGAAGAACTGCTTATAGAC | 3251328-3251348 |  |
| *hyaF*-F | AATGATGAACCGAGTATG | 1024764-1024781 | 91 |
| *hyaF*-F | CGCTTAGTTGATGTTGTA | 1024854-1024837 |  |
| *yccD*-F | GGCTAATGTTACGGTGACT | 1051154-1051136 | 76 |
| *yccD*-R | TTCATTCAACTCCTCTTCAGA | 1051079-1051099 |  |
| *yeaA*-F | GGTGAGTGAAGAATCCATT | 1480536-1480554 | 78 |
| *yeaA*-R | CAGTTACCGCAACGAATT | 1480613-1480596 |  |
| *katE*-F | ATTCCATCCAGGTCATAT | 1530989-1530972 | 83 |
| *katE*-R | TATCGGTATAGGAGAACAA | 1530907-1530925 |  |
| *amyA*-F | AGTTTATCTGGGATTTCAAA | 2002755-2002774 | 133 |
| *amyA*-R | TTACCGAGTTCATCATCA | 2002887-2002870 |  |
| SF2261-F | CGATGACCGTGATATTGA | 2292651-2292668 | 94 |
| SF2261-R | TTTCCTGGTAGTGATTTGT | 2292744-2292726 |  |
| SF1400-F | CGCAGGTATTCAACATGATG | 1440815-1440834 | 79 |
| SF1400-R | CACGGGATAAACTGGTTAATG | 1440893-1440873 |  |
| *hdeA*-F | AACAACAAAGATAAACCAGAAGAT | 3633616-3633593 | 82 |
| *hdeA*-R | TACAAGCCTGAACGATAGC | 3633535-3633553 |  |
| *hyaB*-F | GCAGACTTCATTAACAAC | 1021977-1021994 | 104 |
| *hyaB*-R | AGGACACATTTATCAGAAA | 1022080-1022062 |  |
| *ybaS*-F | AACAACCAACTTCCATAA | 448446-448463 | 120 |
| *ybaS*-R | GTATTGATGAGCGTAGAG | 448565-448548 |  |
| *nmpC*-F | TAAGGAAGGTGCGAACAA | 4029838-4029826 | 94 |
| *nmpC*-R | GCGAAGGTAAGTTGATGAC | 4438147-4438133 |  |
| *rstA*-F | GCAGAACCTTATCGCATTA | 1657447-1657465 | 85 |
| *rstA*-R | CTGATTGTTTATTGTCACTATCC | 1657531-1657509 |  |
| *ykgE*-F | ATCAATAGCGGTTATATCAAAGA | 277820-277798 | 75 |
| *ykgE*-R | ATCGTCGTTATCCTCCAG | 277746-277763 |  |
| *cstA*-F | CTGGGATACATTGCTTTA | 533317-533334 | 83 |
| *cstA*-R | GCGATCAGATAGATACAG | 533399-533382 |  |
| *ybdR*-F | CATTGAACAGGCAGATGATAT | 547997-548017 | 75 |
| *ybdR*-Y | CGATAAAGATGGAGGTCAGA | 548071-548052 |  |
| *gltA*-F | GCGATGTGTTACAAGTAT | 603104-603121 | 86 |
| *gltA*-R | ATCATATTCAGGAAGTTACC | 603189-603170 |  |

^a^Primers were designed according to the genomic sequence of *S. flexneri* 2a 301 (GenBank accession number AE005674). F, forward primer; R, reverse primer.

^b^Location is the locus of the primer in the genomic sequence of *S. flexneri* 2a 301.
