# Supplementary material for: Virulence and Stress Responses of Shigella flexneri Regulated by PhoP/PhoQ
Source: Front Microbiol. 2018 Jan 15;8:2689. doi: 10.3389/fmicb.2017.02689 (PMC5775216; doi:10.3389/fmicb.2017.02689)
Supplement: Table S2 — Homological analysis of Sf301 phoP/phoQ with other homologues. [file Table2.DOCX]

**TABLE S2︱Homological analysis of *Sf*301 *phoP/phoQ* with other homologues.**

| **Strains** | ***Sf*301** | | | |
| --- | --- | --- | --- | --- |
|  | ***phoP* (Num. of nt)** | **Identical%** | ***phoQ* (Num. of nt)** | **Identical%** |
| *S. flexneri 2a str.* 2457*T* | 672 | 100 | 1461 | 100 |
| *S. flexneri 5 str.* 8401 | 672 | 100 | 1461 | 99.9 |
| *S. sonnei Ss*046 | 672 | 98.1 | 1461 | 99.7 |
| *S. dysenteriae Sd*197 | 672 | 98.2 | 1461 | 99 |
| *S. boydii Sb*227 | 672 | 98.1 | 1461 | 99.7 |
| *S. boydii CDC* 3083-94 | 672 | 98.1 | 1461 | 99.7 |
| *E. coli str. K-12 substr. MG*1655 | 672 | 98.8 | 1461 | 99.8 |
| *E.coli O157_H7 str. EDL*933 | 672 | 98.7 | 1383 | 93.6 |
| *S. enterica serovar Typhi str. CT*18 | 675 | 81.6 | 1464 | 76.8 |
| *S. enterica serovar Typhi str. Ty*2 | 675 | 81.6 | 1464 | 76.8 |
| *S. enterica serovar Typhimurium str. LT*2 | 675 | 82.1 | 1464 | 76.5 |
| *S. enterica serovar Enteritidis str. P*125109 | 675 | 81.8 | 1464 | 76.7 |
| *M. tuberculosis H37Ra* | 744 | 44.3 | NE^a^ | / |
| *M. tuberculosis H37Rv* | 744 | 44.6 | NE | / |
| *B. subtilis spizizenii str. W*23 | 723 | 49.2 | NE | / |
| *B. subtilis str.* 168 | 723 | 47.6 | NE | / |
| *S. epidermidis RP62A* | 711 | 47.5 | NE | / |
| *S. aureus MRSA*252 | 702 | 49.4 | NE | / |
| *S. carnosus TM*300 | 717 | 47.1 | NE | / |

NE: not exist.
