# Supplementary material for: Virulence and Stress Responses of Shigella flexneri Regulated by PhoP/PhoQ
Source: Front Microbiol. 2018 Jan 15;8:2689. doi: 10.3389/fmicb.2017.02689 (PMC5775216; doi:10.3389/fmicb.2017.02689)
Supplement: Table S3 — Homological analysis of Sf301 PhoP/PhoQ with other homologues. [file Table3.DOCX]

**TABLE S3︱ Homological analysis of *Sf*301 PhoP/PhoQ with other homologues.**

| **Strains** | ***Sf*301** | | | |
| --- | --- | --- | --- | --- |
|  | **PhoP (Num. of aa)** | **Identical%** | **PhoQ (Num. of aa)** | **Identical%** |
| *S. flexneri 2a str.* 2457*T* | 223 | 100 | 486 | 100 |
| *S. flexneri 5 str.* 8401 | 223 | 100 | 486 | 100 |
| *S. sonnei Ss*046 | 223 | 100 | 486 | 99.8 |
| *S. dysenteriae Sd*197 | 223 | 100 | 486 | 99.4 |
| *S. boydii Sb*227 | 223 | 100 | 486 | 99.8 |
| *S. boydii CDC* 3083-94 | 223 | 100 | 486 | 99.8 |
| *E. coli str. K-12 substr. MG*1655 | 223 | 99.6 | 486 | 99.8 |
| *E.coli O157_H7 str. EDL*933 | 223 | 99.6 | 460 | 93.6 |
| *S. enterica serovar Typhi str. CT*18 | 224 | 99.6 | 487 | 85.2 |
| *S. enterica serovar Typhi str. Ty*2 | 224 | 92.9 | 487 | 85.2 |
| *S. enterica serovar Typhimurium str. LT*2 | 224 | 93.3 | 487 | 85.2 |
| *S. enterica serovar Enteritidis str. P*125109 | 224 | 93.3 | 487 | 85.2 |
| *M. tuberculosis H37Ra* | 247 | 28.6 | NE^a^ | / |
| *M. tuberculosis H37Rv* | 247 | 28.6 | NE | / |
| *B. subtilis spizizenii str. W*23 | 240 | 28.3 | NE | / |
| *B. subtilis str.* 168 | 240 | 28.3 | NE | / |
| *S. epidermidis RP62A* | 236 | 30.5 | NE | / |
| *S. aureus MRSA*252 | 233 | 31.3 | NE | / |
| *S. carnosus TM*300 | 238 | 30.7 | NE | / |

NE: not exist.
