# Supplementary material for: Virulence and Stress Responses of Shigella flexneri Regulated by PhoP/PhoQ
Source: Front Microbiol. 2018 Jan 15;8:2689. doi: 10.3389/fmicb.2017.02689 (PMC5775216; doi:10.3389/fmicb.2017.02689)
Supplement: Table S4 — Primers used for construction of ΔicsA, ΔyoaE, ΔyrbL, ΔrstA and their complementation strain. [file Table4.DOCX]

**TABLE S4︱ Primers used for construction of *△icsA, △yoaE, △yrbL, △rstA* and their complementation strain.**

| **Primers^a^** | **Sequences（5’-3’）** | **Location (bp)^b^** | **Product**  **Length (bp)** | **Annotation^c^** |
| --- | --- | --- | --- | --- |
| **Construction and identification of *△icsA*** | | | | |
| icsAus-kan-F | GAATCTTTTCAGGGGTTTATCAACCACTTACTGATAATATAGTGCGTGTAGGCTGGAGCTGCTTCG | 149599-149643 | 1394 | Underline: up and downstream regions of *icsA*  Wavy line: primers of kanamycin |
| icsAds-kan-R | GAGAAATGCAGGACATCAACACGCCCTGCATTTTTATTATCAGAAATTCCGGGGATCCGTCGACC | 152947-152991 |  |  |
| IntericsA-F | ATGCTATCCACATCACTGGTG | 150617-150637 | 720 |  |
| IntericsA-R | TACGTGTCATAGCTTCAACTG | 151316-151336 |  |  |
| OutericsA-F | TATCGTCCCTTTATTCCGGAT | 149572-149592 | 3437 |  |
| OutericsA-R | TTACACGGTGGGTCCCAGAGA | 152988-153008 |  |  |
| **Construction of *△icsA*c** | | | | |
| *△icsA*c-F | GCGGTCTGAAGCAGACTATCA | 149405-149425 | 3642 |  |
| *△icsA*c-R | CAGGCATACCATCATGTGCAC | 153026-153046 |  |  |
| **Construction and identification of** ***△yoaE*** | | | | |
| yoaEus-kan-F | ATTATTTCTGGCGTCGAATAGCTATTCCTTAAGCAGGAGCTTGTCGTGTAGGCTGGAGCTGCTTCG | 1448669-1448713 | 1394 | Underline: up and downstream regions of *yoaE*  Wavy line: primers of kanamycin |
| yoaEds-kan-R | AATGGACATATCACGCCCATTATGTCGTTACGTTAACGCTTACTCATTCCGGGGATCCGTCGACC | 1450259-1450303 |  |  |
| InteryoaE-F | GACGCCGTCTTCTCGTTGGAT | 1449119-1449139 | 676 |  |
| InteryoaE-R | CATCAACGCCCTCTTCCAGCG | 1449774-1449794 |  |  |
| OuteryoaE-F | GCACTCTATGCCGCACCTTTC | 1448623-1448643 | 1747 |  |
| OuteryoaE-R | CAGCCGATGCCGCTACGTGAT | 1450349-1450369 |  |  |
| **Construction of *△yoaEc*** | | | | |
| *△yoaE*c-F | TCGATTCAGATAAGGGCTACA | 1448324-1448344 | 1979 |  |
| *△yoaE*c-R | ATGGACATATCACGCCCATTA | 1450282-1450302 |  |  |
| **Construction and identification of** ***△yrbL*** | | | | |
| yrbLus-kan-F | ATCGACCATACTGGAGATCGTCAGAAAATATTTCCAGGAGATGGCGTGTAGGCTGGAGCTGCTTCG | 3340574-3340618 | 1394 | Underline: up and downstream regions of *yrbL*  Wavy line: primers of kanamycin |
| yrbLds-kan-R | GTTAGGCGGTGAGCCGTTTATGCAACAACACCAGCTGGATTAAGCATTCCGGGGATCCGTCGACC | 3341246-3341290 |  |  |
| InteryrbL-F | CCATCGTGGCGATGGTGGCGA | 3340717-3340737 | 330 |  |
| InteryrbL-R | CTCACTGATGCGGTGACAGAG | 3341027-3341047 |  |  |
| OuteryrbL-F | AATCACGTACTGAAATCGTTC | 3340458-3340478 | 831 |  |
| OuteryrbL-R | GATTTTACGGCAGATGTATCA | 3341291-3341311 |  |  |
| **Construction of *△yrbLc*** | | | | |
| *△yrbL*c-F | CTTTGACATACAACTCCCTTC | 3340416-3340436 | 827 |  |
| *△yrbL*c-R | TAGGCGGTGAGCCGTTTATGC | 3341268-3341288 |  |  |
| **Construction and identification of** ***△rstA*** | | | | |
| rstAus-kan-F | AACAGCGCGGTGTATTGTGACGTTTTTATATCTACCGTGAATGTTGTGTAGGCTGGAGCTGCTTCG | 1656751-1656795 | 1394 | Underline: up and downstream regions of *rstA*  Wavy line: primers of kanamycin |
| rstAds-kan-R | AATTGAATTCGTTAATTGTTCTCTTTTTCAGATGAACAAATATTAATTCCGGGGATCCGTCGACC | 1657537-1657581 |  |  |
| InterrstA-F | GTCGTGATTTACGCGCAAAGT | 1656986-1657006 | 274 |  |
| InterrstA-R | AGGGTGACTACGCGGTTGATG | 1657239-1657259 |  |  |
| OuterrstA-F | CAGCGCGGTGTATTGTGACGT | 1656753-1656773 | 857 |  |
| OuterrstA-R | GGGTTTGGAATTTGAATAAGC | 1657589-1657609 |  |  |
| **Construction of *△rstAc*** | | | | |
| *△rstA*c-F | ACACCAGGTAAACAAAATAGG | 1656445-1656465 | 1089 |  |
| *△rstA*c-R | ATCTGATTGTTTATTGTCACT | 1657513-1657533 |  |  |

**^a^**Primers were designed according to the genomic sequence of *S. flexneri* 2a 301 (GenBank accession number AE005674). F, forward primer; R, reverse primer.

**^b^**Location is the locus of the primer in the genomic sequence of *S. flexneri* 2a 301.

**^c^**The underlined sequences represent the up and downstream regions of the knockout gene or restriction enzyme sites.
