# Supplementary material for: Virulence and Stress Responses of Shigella flexneri Regulated by PhoP/PhoQ
Source: Front Microbiol. 2018 Jan 15;8:2689. doi: 10.3389/fmicb.2017.02689 (PMC5775216; doi:10.3389/fmicb.2017.02689)
Supplement: Table S5 — Differentially expressed genes of ΔphoPQ compared to Sf301 by microarray and qRT-PCR at early-stationary phase. [file Table5.DOCX]

**TABLE S5︱Differentially expressed genes of *△phoPQ* compared to *Sf*301 by microarray and qRT-PCR at early-stationary phase**

| **Gene** | **Expression ratio (mutant/WT) ^a^** | | | | | **Location** | **Description or predicted function** |
| --- | --- | --- | --- | --- | --- | --- | --- |
|  | **Microarray^b^** | | ***P*** **values^c^** | | **qRT-PCR^d^** |  |  |
| **Amino acid transport and metabolism** | | | | | | | |
| *gntT* | 0.21 | 0.0005 | | ND | | Chromosome | High-affinity transport permease for gluconate |
| *tdcC* | 0.26 | <0.0001 | | 0.52 + 0.13 | | Chromosome | Threonine/serine transporter tdcc |
| *yhaO* | 0.19 | 0.0001 | | ND | | Chromosome | Putative transport system permease protein |
| *tdcB* | 0.29 | 0.0005 | | ND | | Chromosome | Threonine dehydratase |
| *thrC* | 2.15 | 0.0002 | | ND | | Chromosome | Threonine synthase |
| *dppB* | 2.17 | 0.0036 | | ND | | Chromosome | Dipeptide transporter permease dppb |
| *dppA* | 2.23 | 0.0258 | | 1.72 + 0.54 | | Chromosome | Dipeptide transport protein |
| **Carbohydrate transport and metabolism** | | | | | | | |
| *shf* | 0.32 | 0.0003 | | 0.25 + 0.04 | | pCP301 | Putative carbohydrate transport protein |
| *gntK* | 0.38 | 0.0001 | | 0.57 + 0.12 | | Chromosome | Gluconate kinase 1 |
| **Virulence** | | | | | | | |
| *icsA/virG* | 0.45 | <0.0001 | | 0.38 + 0.05 | | pCP301 | Intra- and intercellular Spread, adhesion |
| *virK* | 0.45 | 0.0013 | | 0.35 + 0.06 | | pCP301 | Hypothetical protein |
| **Cell wall/membrane/envelope biogenesis** | | | | | | | |
| *rfbU* | 0.28 | 0.0007 | | 0.33 + 0.08 | | pCP301 | UDP-sugar hydrolase |
| *slyB* | 0.33 | 0.0059 | | 0.21 + 0.06 | | Chromosome | Putative outer membrane protein |
| *mdoB* | 0.45 | 0.0007 | | 0.23 + 0.07 | | Chromosome | Phosphoglycerol transferase I |
| *msbB2* | 0.38 | <0.0001 | | 0.42 + 0.08 | | pCP301 | Lipid A biosynthesis |
| *pmrD* | 0.44 | 0.0170 | | 0.45+ 0.12 | | Chromosome | Polymyxin resistance protein B |
| **Defense mechanisms** | | | | | | | |
| *ybjZ* | 0.38 | 0.0054 | | 0.52 + 0.08 | | Chromosome | Macrolide transporter ATP-binding /permease protein |
| **Energy production and conversion** | | | | | | | |
| *tdcD* | 0.12 | 0.0003 | | ND | | Chromosome | Propionate/acetate kinase |
| *tdcE* | 0.14 | 0.0005 | | ND | | Chromosome | Formate acetyltransferase 3 |
| *gltA* | 2.28 | 0.0207 | | 2.78 + 0.43 | | Chromosome | Type II citrate synthase |
| *fadE* | 2.34 | 0.0029 | | ND | | Chromosome | Acyl-coa dehydrogenase |
| *sdhA* | 2.15 | 0.0228 | | ND | | Chromosome | Succinate dehydrogenase flavoprotein subunit |
| *fumA* | 2.19 | 0.0047 | | ND | | Chromosome | Fumarase A, fumarate hydratase class I; aerobic isozyme |
| *aceA* | 3.01 | <0.0001 | | ND | | Chromosome | Isocitrate lyase |
| **Nucleotide transport and metabolism** | | | | | | | |
| *udp* | 2.09 | 0.0031 | | ND | | Chromosome | Uridine phosphorylase |
| **Posttranslational modification, protein turnover, chaperones** | | | | | | | |
| *ybjX* | 0.06 | 0.0001 | | 0.24 + 0.05 | | Chromosome | Putative enzyme |
| *ibpB* | 0.29 | 0.0008 | | 0.23 + 0.08 | | Chromosome | Heat shock chaperone ibpb |
| *dnaJ* | 0.45 | 0.0004 | | ND | | Chromosome | Chaperone protein dnaj |
| **Signal transduction mechanisms** | | | | | | | |
| *phoP* | 0.002 | <0.0001 | | 0 | | Chromosome | DNA-binding transcriptional regulator phop |
| *phoQ* | 0.003 | <0.0001 | | 0 | | Chromosome | Sensor protein phoq |
| *rstA* | 0.07 | <0.0001 | | 0.12 + 0.02 | | Chromosome | DNA-binding transcriptional regulator rsta |
| *yihK* | 2.21 | 0.0046 | | ND | | Chromosome | GTP-binding protein |
| **Transcription** | | | | | | | |
| *glcC* | 2.51 | 0.0300 | | 2.65 + 0.52 | | Chromosome | DNA-binding transcriptional regulator glcc |
| *gntR* | 0.48 | 0.0255 | | ND | | Chromosome | Regulator of gluconate (gnt) operon |
| **Translation, ribosomal structure and biogenesis** | | | | | | | |
| *yhaR* | 0.11 | 0.0005 | | 0.52 + 0.07 | | Chromosome | Hypothetical protein |
| SF4448 | 0.49 | 0.0212 | | ND | | Chromosome | Trna |
| SF4445 | 0.49 | 0.0129 | | ND | | Chromosome | Trna |
| *rplQ* | 2.08 | 0.0285 | | ND | | Chromosome | 50S ribosomal protein L17 |
| *rplS* | 2.26 | 0.0155 | | 3.22 + 1.24 | | Chromosome | 50S ribosomal protein L19 |
| **General function prediction only** | | | | | | | |
| *yoaE* | 3.79 | 0.0068 | | 5.52 + 1.23 | | Chromosome | Putative transport protein |
| *osmY* | 2.15 | 0.0159 | | ND | | Chromosome | Periplasmic protein |
| **Function unknown** | | | | | | | |
| *yrbL* | 0.02 | <0.0001 | | 0.12 + 0.02 | | Chromosome | Hypothetical protein |
| SF1400 | 0.04 | <0.0001 | | 0.07 + 0.02 | | Chromosome | Hypothetical protein |
| *ycgW* | 0.10 | 0.0001 | | ND | | Chromosome | Hypothetical protein |
| SF2261 | 0.29 | 0.0019 | | 0.15 + 0.04 | | Chromosome | Hypothetical protein |
| SF1401 | 0.33 | 0.0001 | | ND | | Chromosome | Hypothetical protein |
| *yejG* | 2.11 | 0.0165 | | 2.92 + 0.77 | | Chromosome | Hypothetical protein |
| *ygaM* | 2.18 | 0.0001 | | ND | | Chromosome | Hypothetical protein |
| *yhaM* | 0.25 | 0.0013 | | ND | | Chromosome | Hypothetical protein |
| *yicG* | 0.35 | 0.0006 | | ND | | Chromosome | Hypothetical protein |
| *ybfA* | 0.40 | 0.0114 | | ND | | Chromosome | Hypothetical protein |
| SF1773 | 0.48 | 0.0253 | | ND | | Chromosome | Putative acetyltransferase |
| *ychH* | 2.14 | 0.0261 | | ND | | Chromosome | Hypothetical protein |
| *SF4408* | 2.29 | 0.0273 | | ND | | Chromosome | Hypothetical protein |

^a^WT: wild type; ND: not determined.

^b^The differentially expressed genes of microarrays were defined by change ratio> = 2, *P*<0.05.

^c^The *P* values for the DEGs of microarrays.

^d^qRT-PCR data are given as means + standard deviations of results from three independent experiments.
