# Supplementary material for: Virulence and Stress Responses of Shigella flexneri Regulated by PhoP/PhoQ
Source: Front Microbiol. 2018 Jan 15;8:2689. doi: 10.3389/fmicb.2017.02689 (PMC5775216; doi:10.3389/fmicb.2017.02689)
Supplement: Table S6 — The transcriptional levels of phoPQ and its regulated genes in different concentrations of Mg2+ condition. [file Table6.DOCX]

**TABLE S6︱The transcriptional levels of *phoPQ* and its regulated genes in different concentrations of Mg^2+^ condition**

| **Gene** | **qRT-PCR ratio**  **(10 μM Mg^2+^/10 mM Mg^2+^)** | | **Description or predicted function** |
| --- | --- | --- | --- |
|  | ***Sf*301** | ***△phoPQ*** |  |
| *phoP* | 5.05 + 0.19 | / | DNA-binding transcriptional regulator PhoP |
| *phoQ* | 6.16 + 0.61 | / | sensor protein PhoQ |
| *mgtA* | 28.01 + 4.92 | 3.42 + 0.73 | magnesium-transporting ATPase MgtA |
| *slyB* | 2.47 + 1.09 | 1.09 + 0.12 | Cell envelope biogenesis |
| *icsA* | 3.62 + 0.38 | 1.22 + 0.17 | Intra- and intercellular Spread, adhesion |
| *shf* | 6.41 + 0.58 | 1.57 + 0.32 | putative carbohydrate transport protein |
| *virK* | 6.85 + 0.53 | 1.43 + 0.23 | required for proper localization of IcsA (VirG) at the surface of bacteria |
| *yoaE* | 2.55 + 0.1 | 0.89 + 0.12 | Magnesium and cobalt efflux protein |
| *xasA* | 1.81 + 0.16 | 1.21 + 0.08 | acid sensitivity protein, putative transporter |
| *hdeA* | 1.51 + 0.19 | 1.37 + 0.15 | acid-resistance protein |
| *gadA* | 1.03 + 0.03 | 0.82 + 0.09 | glutamate decarboxylase isozyme |
| *yhiW* | 2.98 + 0.15 | 1.04 + 0.12 | putative ARAC-type regulatory protein |
| *virA* | 1.3 + 0.08 | 1.11 + 0.08 | type III secretion protein VirA |
| *ipgA* | 1.23 + 0.13 | 1.09 + 0.14 | chaperone IpgA |
