# Supplementary material for: Virulence and Stress Responses of Shigella flexneri Regulated by PhoP/PhoQ
Source: Front Microbiol. 2018 Jan 15;8:2689. doi: 10.3389/fmicb.2017.02689 (PMC5775216; doi:10.3389/fmicb.2017.02689)
Supplement: Table S7 — The transcriptional levels of phoPQ and its regulated genes in different pH condition. [file Table7.DOCX]

**TABLE S7︱The transcriptional levels of *phoPQ* and its regulated genes in different pH condition**

| **Gene** | **qRT-PCR ratio**  **(pH 5.5/pH 7.0)** | | **Description or predicted function** |
| --- | --- | --- | --- |
|  | ***Sf*301** | ***△phoPQ*** |  |
| *phoP* | 4.53 + 0.59 | / | DNA-binding transcriptional regulator PhoP |
| *phoQ* | 6.17 + 0.94 | / | sensor protein PhoQ |
| *mgtA* | 1.07 + 0.14 | 1.55 + 0.21 | magnesium-transporting ATPase MgtA |
| *slyB* | 1.51 + 0.27 | 1.21 + 0.09 | Cell envelope biogenesis |
| *icsA* | 2.96 + 0.15 | 1.36 + 0.13 | Intra- and intercellular Spread, adhesion |
| *shf* | 3.59 + 0.29 | 1.02 + 0.16 | putative carbohydrate transport protein |
| *virK* | 2.98 + 0.39 | 1.04 + 0.13 | required for proper localization of IcsA (VirG) at the surface of bacteria |
| *yoaE* | 1.17 + 0.15 | 1.32 + 0. 25 | Magnesium and cobalt efflux protein |
| *xasA* | 4.21 + 0.68 | 1.42 + 0.15 | acid sensitivity protein, putative transporter |
| *hdeA* | 7.43 + 1.21 | 2.37 + 0.38 | acid-resistance protein |
| *gadA* | 5.71 + 0.93 | 2.44 + 0.29 | glutamate decarboxylase isozyme |
| *yhiW* | 6.96 + 1.13 | 1.75 + 0.24 | putative ARAC-type regulatory protein |
| *virA* | 0.98 + 0.06 | 1.12 + 0.09 | type III secretion protein VirA |
| *ipgA* | 0.88 + 0.05 | 0.91 + 0.08 | chaperone IpgA |
