# Supplementary material for: Virulence and Stress Responses of Shigella flexneri Regulated by PhoP/PhoQ
Source: Front Microbiol. 2018 Jan 15;8:2689. doi: 10.3389/fmicb.2017.02689 (PMC5775216; doi:10.3389/fmicb.2017.02689)
Supplement: Table S8 — The transcriptional levels of phoPQ and its regulated genes with or without polymyxin B condition. [file Table8.DOCX]

**TABLE S8︱The transcriptional levels of *phoPQ* and its regulated genes with or without polymyxin B condition**

| **Gene** | **qRT-PCR ratio**  **(LB+20 μg/mL polymyxin B /LB)** | | | **Description or predicted function** |
| --- | --- | --- | --- | --- |
|  | ***Sf*301** | ***△phoPQ*** | |  |
| *phoP* | 2.98 + 0.07 | | / | DNA-binding transcriptional regulator PhoP |
| *phoQ* | 3.37 + 0.13 | | / | sensor protein PhoQ |
| *yrbL* | 5.31 + 0.38 | | 1.51 + 0.18 | hypothetical protein |
| *pagP* | 4.38 + 0.18 | | 1.86 + 0.23 | palmitoyl transferase |
| *pmrD* | 7.69 + 0.57 | | 2.45 + 0.33 | polymyxin resistance protein B |
| *msbB2* | 4.42 + 0.19 | | 1.42 + 0.14 | lipid A biosynthesis |
| *slyB* | 4.79 + 0.2 | | 0.88 + 0.12 | Cell envelope biogenesis |
| *mgtA* | 1.95 + 0.53 | | 1.07 + 0.15 | magnesium-transporting ATPase MgtA |
| *icsA* | 7.39 + 0.4 | | 1.33 + 0.23 | Intra- and intercellular Spread, adhesion |
| *shf* | 4.14 + 0.37 | | 1.28 + 0.13 | putative carbohydrate transport protein |
| *yoaE* | 1.07 + 0.51 | | 1.59 + 0.24 | Magnesium and cobalt efflux protein |
| *virA* | 1.09 + 0.21 | | 1.37 + 0.21 | type III secretion protein VirA |
